# Gut-brain peptides in corticostriatal-limbic circuitry and alcohol use disorders

**DOI:** 10.3389/fnins.2014.00288

**Published:** 2014-09-18

**Authors:** Chelsea A. Vadnie, Jun Hyun Park, Noha Abdel Gawad, Ada Man Choi Ho, David J. Hinton, Doo-Sup Choi

**Affiliations:** ^1^Department of Molecular Pharmacology and Experimental Therapeutics, Mayo Clinic College of MedicineRochester, MN, USA; ^2^Neurobiology of Disease Program, Mayo Clinic College of MedicineRochester, MN, USA; ^3^Department of Psychiatry, Sanggye Paik Hospital, College of Medicine, InJe UniversitySeoul, South Korea; ^4^Department of Psychiatry and Psychology, Mayo Clinic College of MedicineRochester, MN, USA

**Keywords:** peptide, alcohol, GLP-1, ghrelin, substance P, neurotensin, NPY

## Abstract

Peptides synthesized in endocrine cells in the gastrointestinal tract and neurons are traditionally considered regulators of metabolism, energy intake, and appetite. However, recent work has demonstrated that many of these peptides act on corticostriatal-limbic circuitry and, in turn, regulate addictive behaviors. Given that alcohol is a source of energy and an addictive substance, it is not surprising that increasing evidence supports a role for gut-brain peptides specifically in alcohol use disorders (AUD). In this review, we discuss the effects of several gut-brain peptides on alcohol-related behaviors and the potential mechanisms by which these gut-brain peptides may interfere with alcohol-induced changes in corticostriatal-limbic circuitry. This review provides a summary of current knowledge on gut-brain peptides focusing on five peptides: neurotensin, glucagon-like peptide 1, ghrelin, substance P, and neuropeptide Y. Our review will be helpful to develop novel therapeutic targets for AUD.

## Introduction

### Gut-brain peptides control food and alcohol intake

Peptides secreted by endocrine cells and enteric neurons in the gastrointestinal (GI) tract and neurons in the central nervous system (CNS), have been implicated in the pathophysiology of alcohol use disorders (AUD). Gut-brain peptides have different functions when administered into the brain relative to the periphery since the blood-brain barrier (BBB) restricts access of peripheral peptides to the CNS. In addition, gut-brain peptides typically have a short half-life due to rapid inactivation by peptidases. Although gut-brain peptides have numerous site-specific functions, they are generally considered to act as regulators of energy intake and expenditure. Alcohol is both an addictive substance and a source of energy. Therefore, it is not surprising that there are peptidergic signaling links between appetitive- and alcohol-related behaviors.

There are numerous lines of evidence indicating a mechanistic link between alcohol drinking and food intake. For example, there is a high comorbidity between AUD and eating disorders, especially bulimia (Goldbloom et al., 1992; Dansky et al., 2000; Bulik et al., 2004). Interestingly, many approved and experimental treatments for AUD (including naltrexone, disulfiram, baclofen, and varenicline) suppress appetite and reduce body weight (Leggio et al., 2011). Furthermore, the hedonic response to sucrose in humans was shown to associate with parental history of AUD, suggesting that hedonic response to sucrose may indicate a genetic risk for AUD (Kampov-Polevoy et al., 2001, 2003). Sweet preference is partially regulated by gut-brain peptide signaling in the brain. Thus, the same gut-brain peptides that regulate hedonic food intake may also affect alcohol consumption (Furudono et al., 2006; Skibicka et al., 2011). As discussed in this review, many gut-brain peptides and their receptors are expressed within corticostriatal-limbic circuitry. Since there is overlapping corticostriatal-limbic circuitry that regulates food intake and alcohol consumption (Volkow et al., 2009, 2012b; Kenny, 2011), it is not surprising that some orexigenic peptides (ghrelin) increase (Schneider et al., 2007; Jerlhag et al., 2009) and some anorexigenic peptides [neurotensin, glucagon-like peptide 1 (GLP-1)] decrease alcohol consumption (Lee et al., 2010; Egecioglu et al., 2012). However, alcohol has direct pharmacological effects on neurons that differ from the effects of palatable food intake, which may explain why there are also examples of gut-brain peptides [e.g., neuropeptide Y (NPY)] that exhibit opposite effects on feeding and alcohol drinking (Thorsell et al., 2005a).

### Corticostriatal-limbic circuitry and AUD

**Figure 1:**
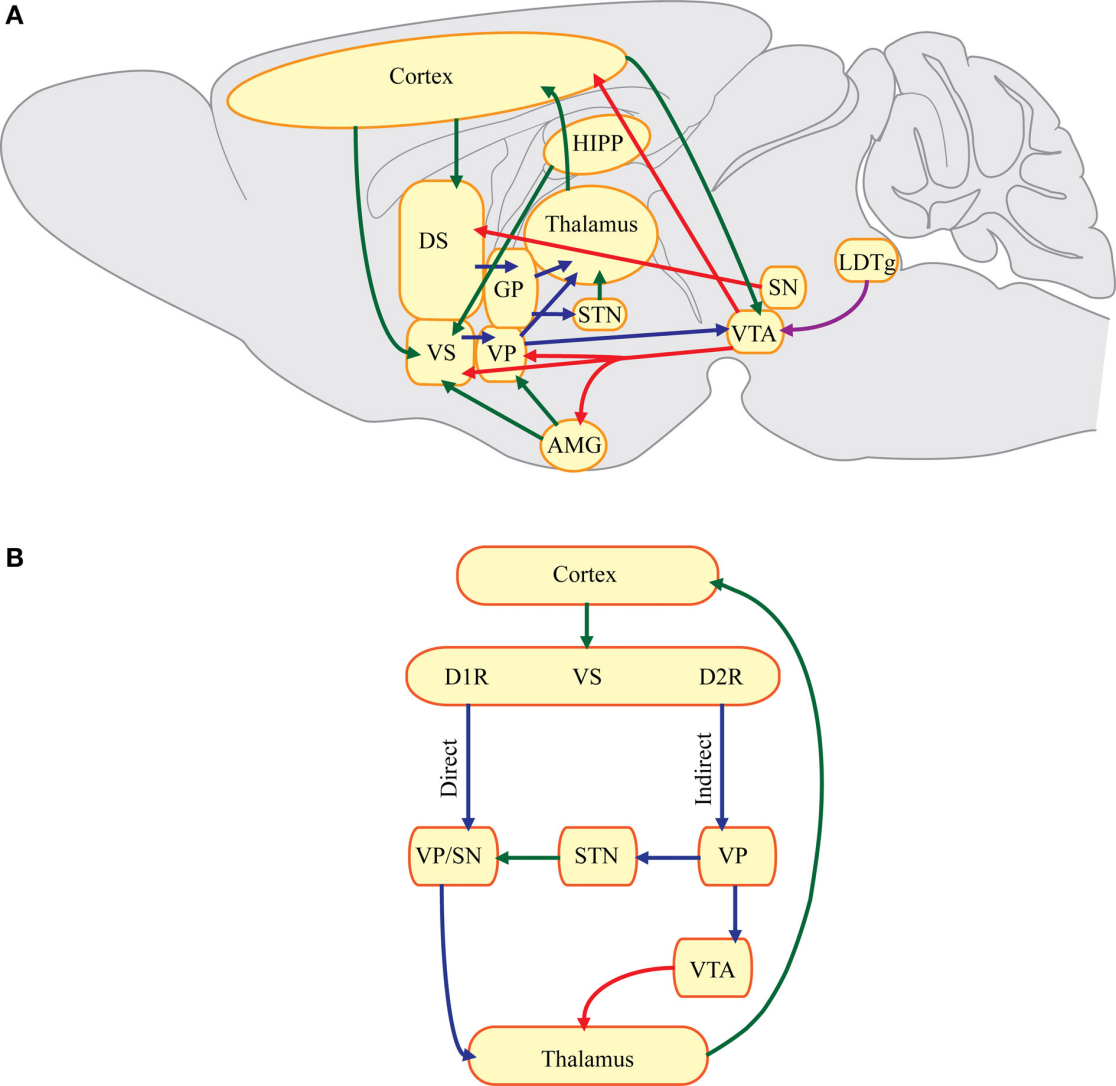
**Simplified schematic of corticostriatal-limbic circuitry. (A)** Key corticostriatal-limbic connections where gut-brain peptides may act to influence addictive behaviors. AMG, amygdala; DS, dorsal striatum; GP, globus pallidus; HIPP, hippocampus; LDTg, laterodorsal tegmental area; SN, substantia nigra; STN, subthalamic nucleus; VP, ventral pallidum; VS, ventral striatum; VTA, ventral tegmental area. **(B)** A hypothetical schematic of the direct and indirect pathways in the VS. Glutamatergic projections from the cortex synapse onto MSNs expressing predominantly dopamine D1 receptors (D1R) or dopamine D2 receptors (D2R). MSNs expressing D1R may project directly to the VP or the substantia nigra (SN) pars reticulata forming the direct pathway. Inhibition of GABAergic neurons in the VP or SN can then disinhibit downstream thalamocortical targets. MSNs expressing dopamine D2 receptors project to the VP which synapses onto glutamatergic neurons in the STN. Therefore, activation of the indirect pathway results in reduced glutamatergic projections to the SN pars reticulata. Another possible indirect pathway involves projections from the VP to dopaminergic neurons in the VTA. The VTA may inhibit downstream thalamocortical targets. Green, glutamatergic fibers; red, dopaminergic fibers; blue, GABAergic fibers; purple, cholinergic fibers.

Many gut-brain peptides and/or their receptors are found in corticostriatal-limbic circuitry, where alcohol-induced maladaptations are thought to underlie the behaviors associated with AUD (Binder et al., 2001a; Korotkova et al., 2003; Herpfer and Lieb, 2005; Dickson et al., 2011; Volkow et al., 2012a; Egecioglu et al., 2013; Shirazi et al., 2013). As shown in Figure 1A, the nucleus accumbens (NAc, part of the ventral striatum) and dorsal striatum receive glutamatergic projections from the cortex. However, the NAc also receives glutamatergic projections from the hippocampus and amygdala. Dopaminergic afferents from the ventral tegmental area (VTA) in the midbrain project largely to the NAc (mesolimbic pathway), while the substantia nigra (SN) pars compacta is the main source of dopamine in the dorsal striatum (nigrostriatal pathway). GABAergic medium spiny neurons (MSNs) in the striatum are the primary output neurons that are typically quiescent. When these neurons are activated, they tend to inhibit the tonically active pallidum, which contains primarily GABAergic neurons (Graybiel, 2000). Therefore, MSNs in this “direct pathway” or striatonigral pathway disinhibit thalamocortical targets, which facilitates movement and possibly drug seeking. In contrast, MSNs in the “indirect pathway” or the striatopallidal pathway inhibit downstream thalamocortical targets and activation of this pathway may suppress certain behaviors (Graybiel, 2000). The direct and indirect pathways are traditionally described in reference to dorsal striatal circuitry, but similar pathways are thought to exist in in the NAc (Figure 1B) (Sesack and Grace, 2010). Corticostriatal-limbic circuitry is important for selecting and amplifying behaviors (Yin and Knowlton, 2006). Thus, parallel and serial connections within corticostriatal-limbic circuitry are thought to underlie various phenotypes associated with the stages of AUD.

**Figure 2:**
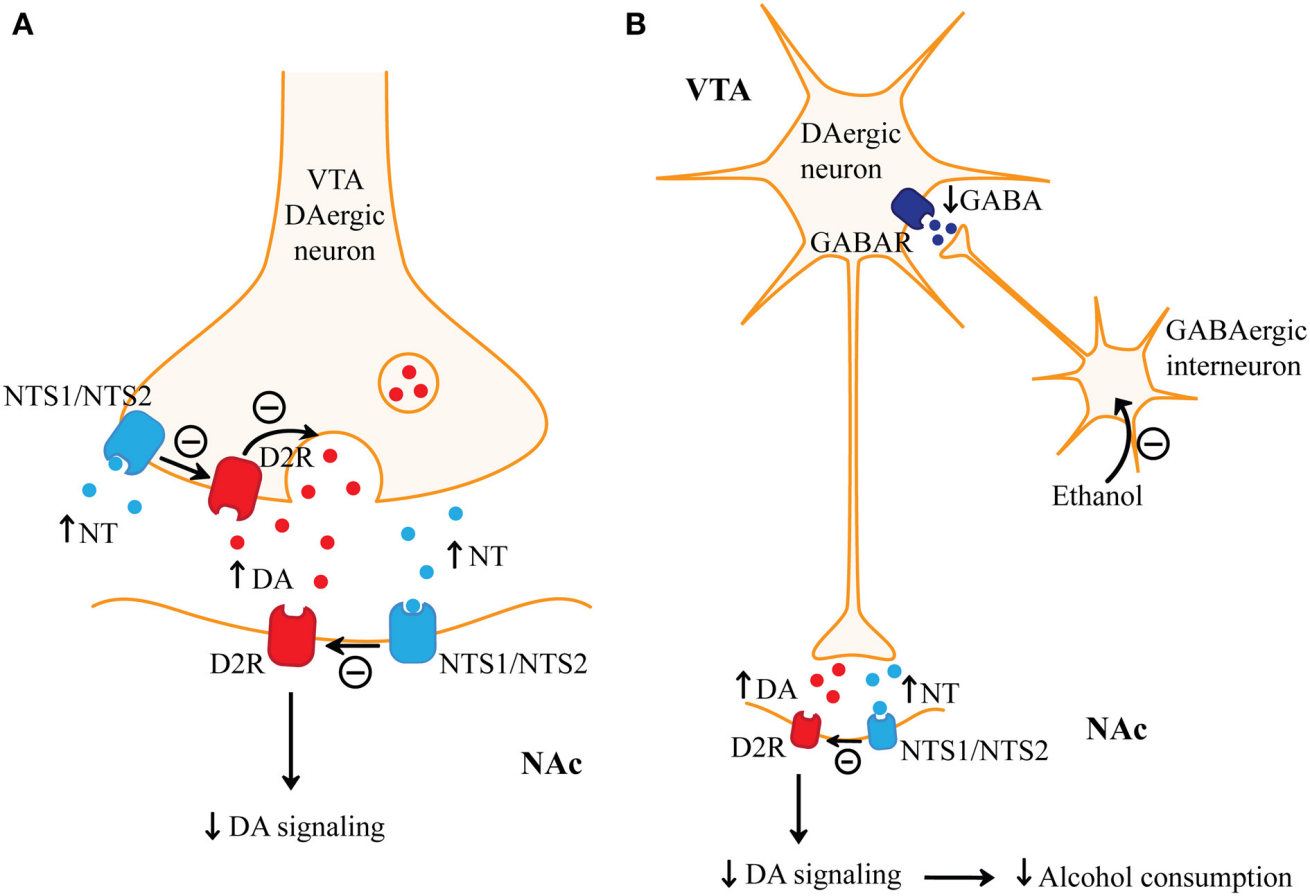
**Regulation of alcohol consumption by neurotensin. (A)** Neurotensin (NT) has opposing presynaptic and postsynaptic effects on dopaminergic signaling in the nucleus accumbens (NAc). Presynaptically, NT may increase dopamine (DA) release through neurotensin receptor type 1 (NTS1)-, or possibly even neurotensin receptor type 2 (NTS2)-mediated inhibition of dopamine D2 receptors (D2R) on ventral tegmental area (VTA) dopaminergic (DAergic) terminals. Postsynaptically, NTS1 or even NTS2 dimerizes with D2R and attenuates D2R signaling. Functional studies suggest that the effect of NT on inhibiting postsynaptic D2R signaling may predominate in the nucleus accumbens (NAc). **(B)** Alcohol increases dopamine release in the NAc. Alcohol may excite VTA dopaminergic neurons by inhibiting GABAergic interneurons in the VTA, which results in reduced activation of GABA receptors (GABAR) on VTA dopaminergic neurons. Increased VTA-mediated dopamine in the NAc may promote alcohol consumption and mediate alcohol reward. NT receptor agonists may reduce alcohol consumption by acting on postsynaptic NT receptors which may inhibit alcohol-mediated dopamine signaling in the NAc.

**Figure 3:**
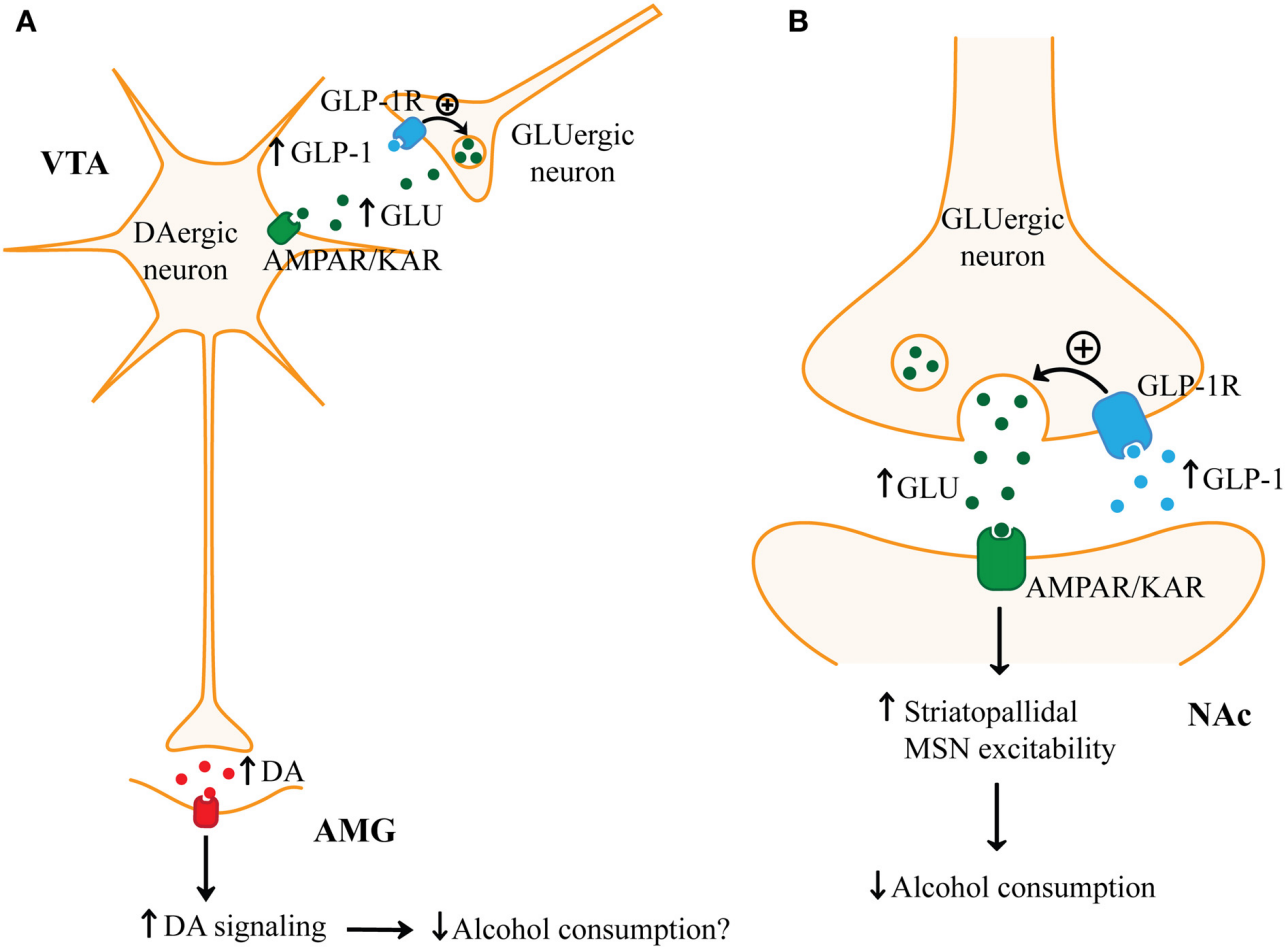
**Possible mechanisms by which GLP-1 may reduce alcohol consumption. (A)** Glucagon-like peptide 1 (GLP-1), by acting on the GLP-1 receptor (GLP-1R) on presynaptic glutamatergic (GLUergic) terminals in the ventral tegmental area (VTA), may increase glutamate (GLU) release. Increased glutamate release in the VTA may enhance the activity of VTA dopaminergic (DAergic) neurons through glutamatergic AMPA receptors (AMPARs) or kainate receptors (KARs). Increased activity of VTA dopaminergic neurons may then promote dopamine release in postsynaptic targets such as the amygdala (AMG). Increased dopamine D2 receptor (D2R) signaling in the amygdala has been linked with decreased food intake and therefore may mechanistically explain how GLP-1 decreases alcohol consumption. **(B)** GLP-1 through GLP-1R on presynaptic glutamatergic terminals may increase glutamate release in the nucleus accumbens (NAc). Increased NAc glutamate may activate AMPARs or KARs which may reduce alcohol consumption by enhancing the activity of striatopallidal medium spiny neurons (MSNs).

AUD can be separated into three stages: (1) binge/intoxication, (2) preoccupation/anticipation, (3) withdrawal/negative affect (Koob and Volkow, 2010). The binge/intoxication stage refers to initial alcohol consumption. Increased sensitivity to the intoxicating effects often deters consumption, while increased sensitivity to the rewarding effects of alcohol may promote intake (King et al., 2011). Alcohol reward seems to be primarily mediated by the alcohol-induced dopamine response in the NAc. Alcohol enhances the firing of the VTA, which projects to the NAc, and this is associated with the perception of alcohol reward (Gessa et al., 1985; Volkow et al., 2009). Here, we discuss how neurotensin has potential for reducing baseline consumption through enhancing sensitivity to the intoxicating effects of alcohol and possibly reducing alcohol reward (Figure 2). In addition, GLP-1 may suppress heavy drinking by reducing the rewarding effects of alcohol (Figure 3). During the early stages of AUD, positive reinforcement likely plays a major role. The transition from social or impulsive alcohol use to uncontrolled compulsive drinking is hypothesized to involve increased recruitment and plasticity of corticostriatal-limbic circuitry (Koob and Volkow, 2010). Initially, the ventral circuitry and prefrontal cortex (PFC) plays a greater role, then in the later stages of AUD the dorsal striatum is recruited (Everitt et al., 2008). The dorsal striatum can be divided into the dorsomedial (DMS, caudate) and dorsolateral (DLS, putamen) striatum. The DMS has been linked to goal-directed behavior, while the DLS has been associated with habitual behavior (Yin and Knowlton, 2006). During the development of AUD there may be a shift of control from the DMS to DLS as drinking becomes more compulsive. Some gut-brain peptides or their receptors are found in the dorsal striatum, but whether they influence addictive behaviors through altering dorsal striatal function is an area that needs to be explored.

**Figure 4:**
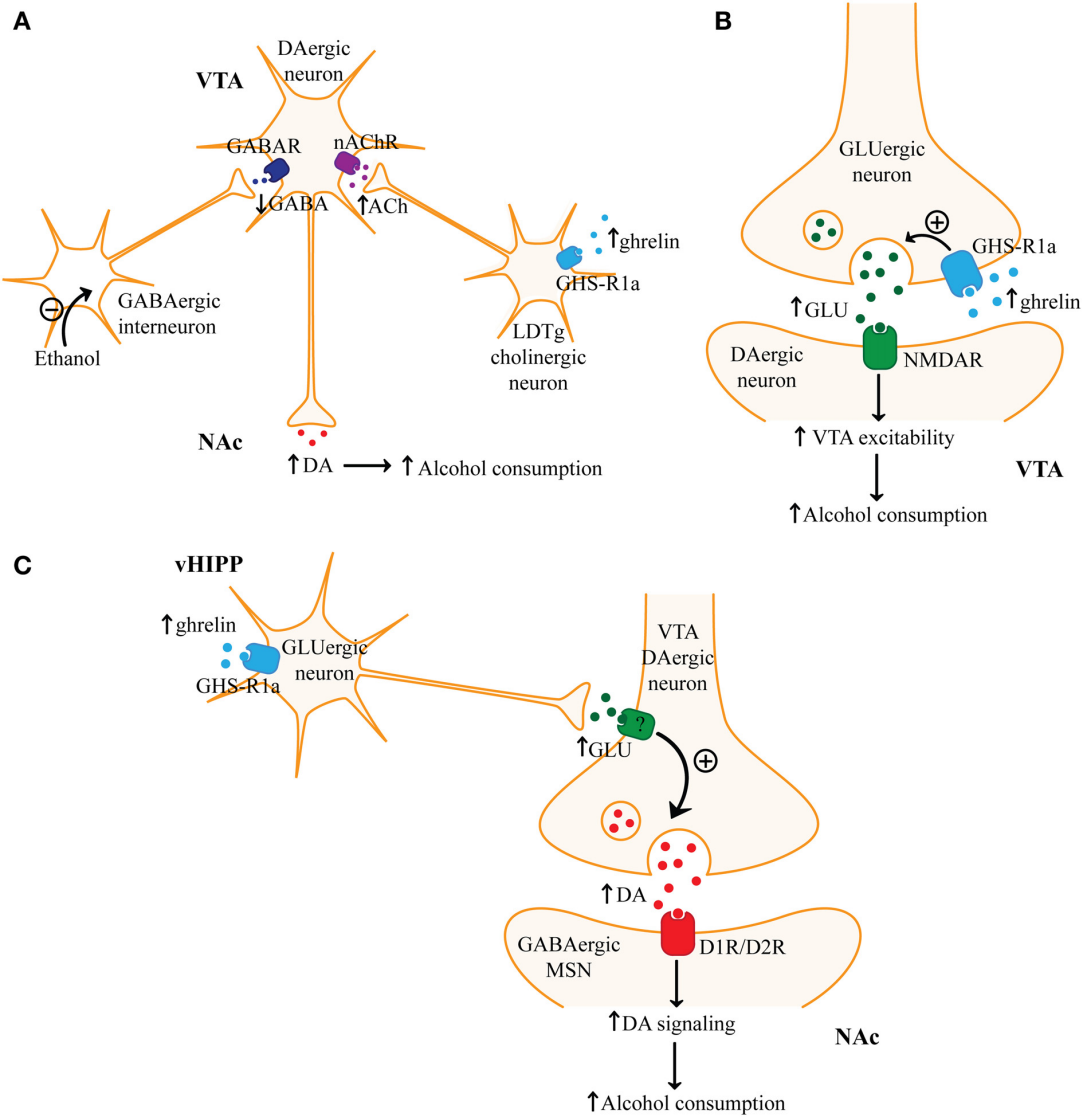
**Ghrelin signaling and alcohol consumption. (A)** One mechanism by which ghrelin may increase ventral tegmental area (VTA) dopaminergic neuron activity and nucleus accumbens (NAc) dopamine (DA) levels involves ghrelin-mediated activation of laterodorsal tegmental area (LDTg) cholinergic neurons. Ghrelin through growth hormone secretagogue receptor 1a (GHS-R1a) may activate LDTg cholinergic neurons and subsequently increase acetylcholine (ACh) levels in the VTA. Activation of nicotinic acetylcholine receptors (nAChR) on VTA dopaminergic neurons can increase neuronal activity and promote dopamine release in postsynaptic regions. Increased dopamine signaling may explain how ghrelin can promote alcohol consumption. **(B)** There is also evidence that ghrelin may activate VTA dopaminergic neurons by promoting presynaptic glutamate (GLU) release in the VTA. Ghrelin through GHS-R1a on glutamatergic terminals in the VTA may enhance glutamate release. Subsequent activation of NMDA receptors (NMDAR) could then increase VTA dopaminergic activity. **(C)** Finally, ghrelin through GHS-R1a may activate glutamatergic ventral hippocampal (vHIPP) neurons, which may result in increased glutamate release in the NAc. Increased glutamate in the NAc may increase dopamine release by activating glutamate receptors on presynaptic dopaminergic terminals. Increased activation of dopamine D1 or D2 receptors (D1R/D2R) on medium spiny neurons (MSNs) in the NAc may then promote alcohol consumption.

The preoccupation/anticipation stage, or the craving stage, often results in relapse and is perhaps the most challenging aspect of AUD treatment. Conditioned reinforcement or pairing of stimuli with the effects of alcohol, contributes to craving for alcohol (Weiss, 2005). Conditioned reinforcers, which were once neutral stimuli, can increase dopamine on their own and signal expectation of reward (Saunders and Robinson, 2012). Craving and increased motivation to seek alcohol often occur after reexposure to alcohol or an alcohol-associated cue, which may induce relapse or reinstatement. The NAc, as well as the cortical and limbic sites that project to the NAc, are key sites for conditioned reinforcement and relapse (Weiss, 2005). Here, we discuss evidence that suggests that ghrelin receptor antagonists may effectively reduce alcohol craving (Figure 4).

**Figure 5:**
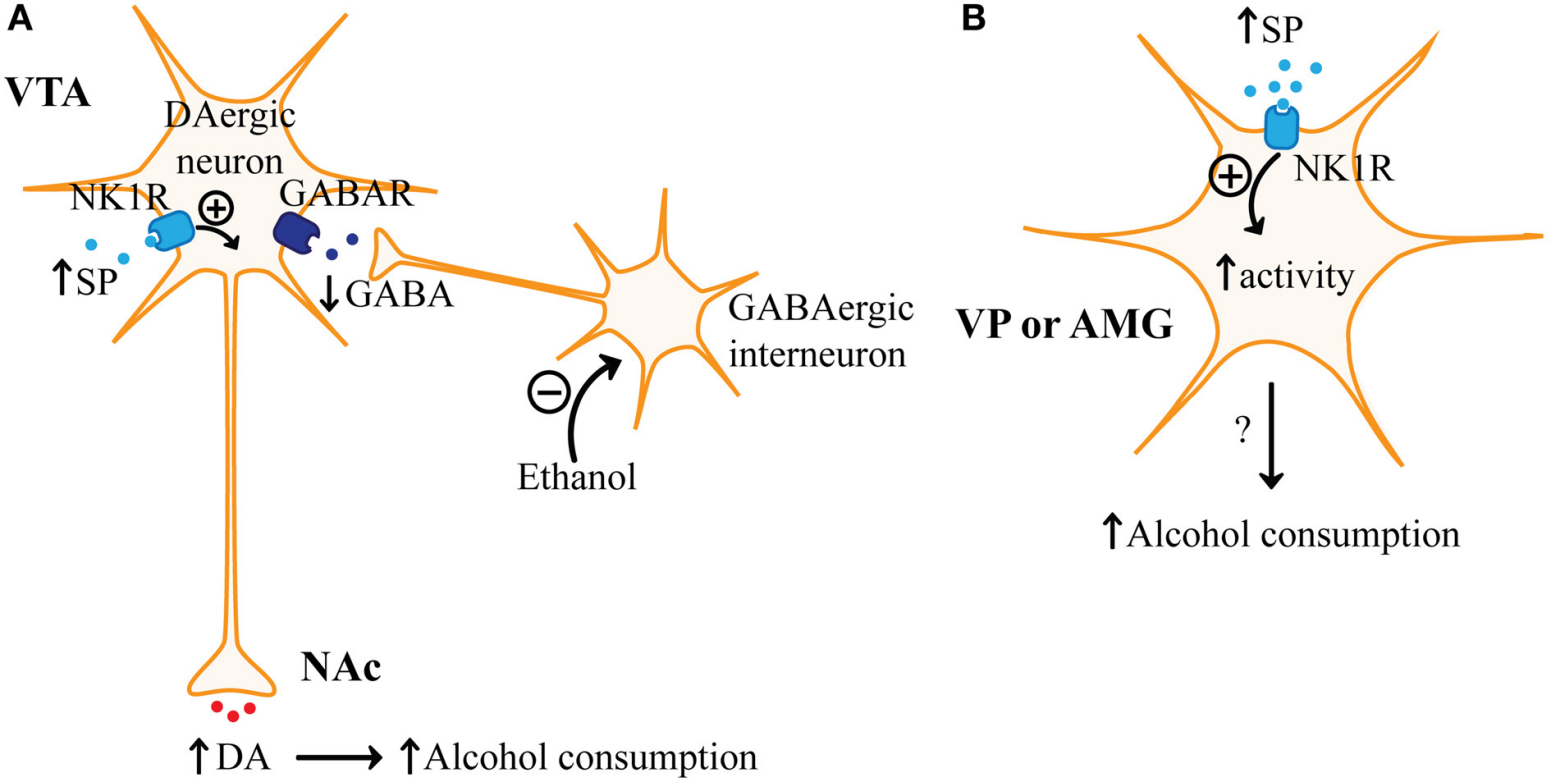
**Regulation of alcohol consumption by substance P. (A)** Increased levels of substance P (SP) released from neurons or by microinjection of SP into the ventral tegmental area (VTA) interacts with neurokinin receptor 1 (NKR1) on VTA dopaminergic (DAergic) neurons resulting in increased release of dopamine (DA) to areas like the nucleus accumbens (NAc). Increased dopamine release in the NAc, as a result of increased SP in the VTA, would be expected to increase alcohol consumption since alcohol is known to disinhibit VTA dopaminergic neurons. Alcohol is thought to inhibit GABAergic interneurons in the VTA, which results in reduced activation of GABA receptors (GABAR) on VTA dopaminergic neurons. **(B)** NK1R is also found in the ventral pallidum (VP) and the amygdala (AMG). SP may increase administration of rewarding substances like alcohol by increasing the excitability of neurons in the VP and AMG.

**Figure 6:**
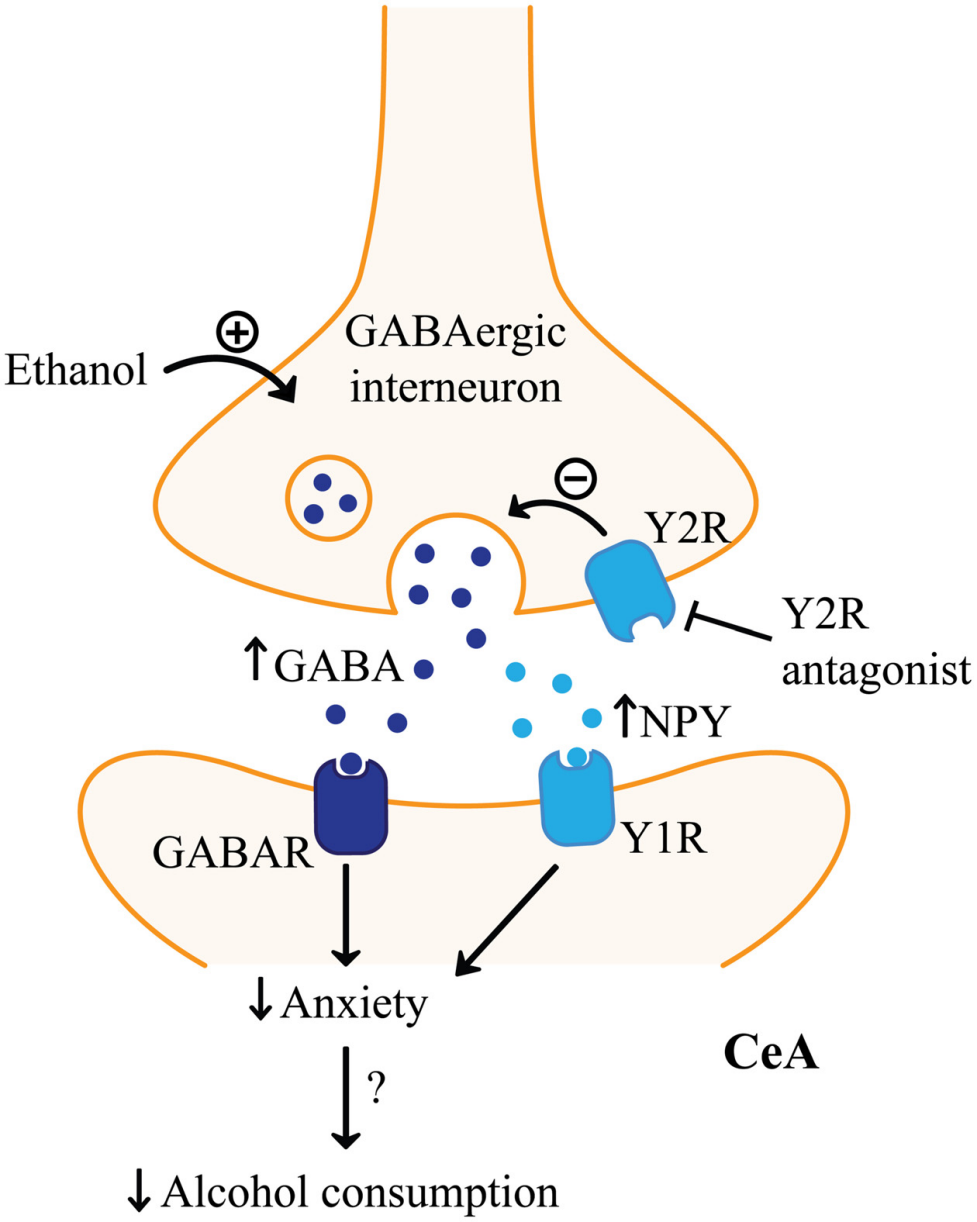
**NPY signaling in the amygdala and alcohol consumption**. Neuropeptide Y (NPY) receptor type 1 (Y1R) agonists and NPY receptor type 2 (Y2R) antagonists in the central amygdala (CeA) may reduce alcohol consumption through mimicking the effects of acute alcohol on GABAergic signaling. Both receptors are thought to be inhibitory, but differ in their location. Y2R may be predominantly on presynaptic GABAergic interneurons, where Y1R may be expressed postsynaptically on GABAergic output neurons. Therefore, inhibiting Y2R function would increase GABA release from GABAergic interneurons in the CeA, similar to alcohol which is known to facilitate GABAergic signaling in the amygdala. Increased activation of GABA receptors (GABAR) in the CeA would reduce CeA output. Activating Y1R would also inhibit postsynaptic CeA neurons similar to increasing GABAergic signaling. Furthermore, Y2R antagonists would be expected to increase NPY which would facilitate the effect at Y1R. Decreased CeA output has been linked with reduced anxiety which is hypothesized to suppress stress-induced alcohol relapse.

The withdrawal/negative affect stage predominates after numerous cycles of alcohol intoxication and withdrawal. Tolerance develops to the intoxicating effects of alcohol, but higher, more frequent doses of alcohol can generate more severe withdrawal symptoms (Goldstein, 1972). Alcohol consumption may suppress feelings of dysphoria and anxiety, which can further escalate alcohol drinking (Gilman et al., 2008). The amygdala appears to regulate negative affect related to alcohol withdrawal (Koob, 2013). Excessive glutamate in the PFC and striatum has also been linked to withdrawal symptoms (Hinton et al., 2012; Bauer et al., 2013). Here, we discuss how substance P (Figure 5) and NPY (Figure 6) are promising targets for reducing especially stress-mediated relapse.

### Animal models for AUD

Animal models have been essential to advance AUD research. Inbred mouse strains, with an essentially homozygous genetic background at every locus, have been useful to study the underlying molecular mechanisms of AUD. C57BL/6J inbred mice are known to drink the most alcohol whereas DBA/2J inbred mice drink very little alcohol (Belknap et al., 1993; Moore et al., 2010). C57BL/6J x DBA/2J recombinant inbred strains have been used to identify the genetics behind addictive behaviors (Cunningham, 1995). Another technique for generating animal models for AUD is selective breeding of high alcohol-preferring rats or mice. Here we will discuss findings related to gut-brain peptides in Indiana University alcohol-preferring (P) rats (vs. alcohol-non-preferring, NP), alcohol-accepting (AA) rats (vs. alcohol-non-accepting, ANA), high-alcohol-drinking (HAD) rats (vs. low-alcohol-drinking, LAD) and Warsaw high-preferring (WHP) rats (vs. Warsaw low-preferring, WLP). Although these rats have all been bred to have the same phenotype, i.e., high alcohol consumption, they differ in their genetic background. For example, P rats are derived from outbred Wistar rats, whereas the HAD line is derived from N/Nih rats (Li et al., 1979, 1993). P and NP rats are commonly used since they model many aspects of AUD, such as binge- and relapse-like drinking (Bell et al., 2006). P rats exhibit anxiety-like behavior and are sensitive to the anxiolytic effects of alcohol (Stewart et al., 1993). Mice have also been selectively bred for their sensitivity to alcohol-induced loss of righting reflex or the hypnotic effect of alcohol. These long-sleep (LS) and short-sleep (SS) mice and recombinant inbred LL × SS lines have been used to examine the genetics behind differences in sensitivity to the intoxicating effects of alcohol (Bennett et al., 2006). Lastly, specific genetic effects on alcohol-related behaviors can be studied by knockout, knockin, and transgenic mice.

There are numerous different behavioral paradigms that are used to model AUD-like behaviors in animals. Baseline alcohol consumption resembling the binge/intoxication stage of AUD is often measured in animals by two-bottle choice drinking (continuous, limited or intermittent access), operant self-administration or binge-like drinking-in-the-dark (Samson et al., 1988; Belknap et al., 1993; Rhodes et al., 2005). Limited and intermittent access paradigms are especially valuable since they appear to better model escalated alcohol consumption seen in humans, and the transition to other stages of AUD, such as withdrawal (Hwa et al., 2011). To investigate sensitivity to alcohol-induced sedation and ataxia in animals, performance on the rotarod and alcohol-mediated loss of righting reflex is often examined (Crabbe et al., 1981; Dar, 1996). Alcohol-mediated conditioned place preference (CPP) and brain stimulation reward thresholds are typically used to evaluate alcohol reward sensitivity (Kornetsky, 1985; Cunningham et al., 2006). Anticipation for, or motivation to, consume alcohol in animals (similar to the preoccupation/anticipation stage) is often measured by progressive ratio operant responding where the breakpoint, or the maximum number of responses for a single reward, is determined (Stafford et al., 1998). Alcohol preoccupation/anticipation may lead to relapse which can be studied in animals by assessing drug- and cue-induced reinstatement (Sanchis-Segura and Spanagel, 2006). Furthermore, negative affect related to alcohol withdrawal can be examined in animals by conditioned place aversion and anxiety measures (e.g., elevated plus maze, time in the center of the open field) (Valdez et al., 2002; Prut and Belzung, 2003; Cunningham et al., 2006). The severity of alcohol withdrawal is often quantified by handling-induced convulsions in rodents (Metten and Crabbe, 2005). Stress and anxiety outside of alcohol withdrawal may also promote relapse which can be assessed in rodents by quantifying alcohol reinstatement after a pharmacological stressor (yohimbine), for example (Marinelli et al., 2007). In this review, we will discuss how several gut-brain peptides may influence AUD-related behaviors as demonstrated in animal and human studies.

## Neurotensin

Neurotensin (NT) is a 13-amino-acid (a.a.) peptide originally isolated from the bovine hypothalamus (Carraway and Leeman, 1973). NT is expressed in many tissues both centrally and peripherally. In the CNS, NT is widely expressed at high levels in the amygdala, hypothalamus, SN and VTA, and at lower levels in areas such as the hippocampus, cortex and striatum (Manberg et al., 1982; Stolakis et al., 2010; Boules et al., 2013). Thus, NT is found within corticostriatal-limbic circuitry where it acts as a neurotransmitter and neuromodulator (Binder et al., 2001a; Ferraro et al., 2008; Petkova-Kirova et al., 2008a,b). Centrally, NT has a wide range of actions. NT plays a role in opioid-independent antinociception (Kleczkowska and Lipkowski, 2013), hypothermia (Bissette et al., 1976), blood pressure (Sumners et al., 1982), anterior pituitary hormone secretion (Rostene and Alexander, 1997; Stolakis et al., 2010), food intake (Cooke et al., 2009), and sleep (Fitzpatrick et al., 2012). NT also possesses antipsychotic-like effects, which are largely attributed to NT-mediated regulation of dopaminergic signaling (Binder et al., 2001b; Kinkead and Nemeroff, 2004, 2006; Vadnie et al., 2014). The neuromodulatory role of NT on dopamine signaling also makes NT an attractive target for substance abuse research.

NT exerts its effects by acting on three receptor subtypes (NTS1, NTS2, and NTS3; Vincent et al., 1999). NTS1 and NTS2 are G-protein-coupled receptors through which NT primarily exerts its effects. NTS1 has the highest affinity for NT and is broadly expressed in the CNS (Pelaprat, 2006). NTS1 is found in areas such as the SN, VTA, NAc, hypothalamus, amygdala, PFC, hippocampus, and dorsal root ganglia of the spinal cord (Alexander and Leeman, 1998; Fassio et al., 2000). NTS2 is localized to many of the same brain regions as NTS1 (Mazella et al., 1996; Walker et al., 1998; Sarret et al., 2003). However, there are especially high levels of NTS2 in areas associated with pain, where NT agonists have been shown to regulate pain through NTS2 independent of NTS1 (Dubuc et al., 1994; Boules et al., 2010).

### Neurotensin and corticostriatal-limbic circuitry

NT and NTS1 are highly expressed in the mesocorticolimbic dopamine pathways (Binder et al., 2001a) where NT regulates dopamine transmission mainly through NTS1. NT-mediated activation of NTS1 is known to inhibit dopamine D2 receptor (D2R) function through a direct NTS1/D2 receptor–receptor interaction which results in a reduced affinity of D2R for dopamine (Figure 2A; Koschatzky et al., 2011). NT may promote the internalization of D2R and may reduce D2R signaling (Jomphe et al., 2006; Thibault et al., 2011; Borroto-Escuela et al., 2013). Conversely, NT or NT analogs increased tyrosine hydroxylase gene expression and dopamine release, which may be mediated by NTS1 on dopaminergic neurons (Kalivas and Duffy, 1990; Burgevin et al., 1992; Wang et al., 2004; Jomphe et al., 2006). The opposing presynaptic and postsynaptic effects of NTS1 on striatal dopaminergic signaling complicate our understanding of NT's role in the modulation of striatal-mediated addictive behaviors. When NT is injected into the NAc, it reduces the response to psychostimulants (Ervin et al., 1981; Robledo et al., 1993). Conversely, higher concentrations of NT in the NAc increase dopamine release, which may be mediated by NTS1 on presynaptic dopaminergic terminals, and would be predicted to enhance the effects of psychostimulants (Figure 2A) (Reyneke et al., 1990; Chapman et al., 1992). Immunohistochemistry and behavioral studies suggest that in the dorsal striatum there is a higher density of NTS1 on dopaminergic terminals relative to postsynaptic MSNs (Ervin et al., 1981; Li et al., 1995; Boudin et al., 1996; Peltonen et al., 2012). Systemic administration data argue that the postsynaptic antagonistic effects of NTS1 on D2R function may predominate since brain-penetrating NT analogs given systemically reduce the pharmacological effects of psychostimulants and do not appear to be rewarding (Binder et al., 2001b; Fantegrossi et al., 2005). Thus, NTS1 activation may modulate alcohol-related behaviors by inhibiting D2R signaling in striatopallidal MSNs in the NAc (Figure 2B), but not in the dorsal striatum where stimulation of NTS1 may promote dopamine signaling.

Recently, an *in vitro* study showed that in the presence of NT and NTS2, D2R exhibits a reduced affinity for dopamine similar to NTS1 (Koschatzky and Gmeiner, 2012). In addition, the NTS2 selective agonist NT79 was shown to block amphetamine-mediated hyperactivity like NTS1 agonists (Boules et al., 2010). These studies suggest that NTS2 may also inhibit dopamine signaling (Figure 2A). Beyond these studies, the role of NTS2 in regulating addictive behaviors through dopamine signaling has been relatively unexplored.

NT may also regulate glutamate transmission in corticostriatal-limbic circuitry. The NT analog NT69L inhibited alcohol-mediated glutamate release in the striatum (Li et al., 2011). Alcohol has a biphasic effect on striatal extracellular glutamate levels with low doses increasing and high doses decreasing extracellular glutamate (Moghaddam and Bolinao, 1994; Chen et al., 2010). This suggests that NTS1 or NTS2 activation may inhibit the acute effects of low doses of alcohol in the striatum. However, the mechanism behind this effect is unclear since other studies have suggested that NT alone promotes glutamatergic signaling (Ferraro et al., 2011). NTS1 has been shown to functionally interact with glutamatergic NMDA receptors, potentiating NMDA receptor function (Ferraro et al., 2012). Alcohol is a known to inhibit NMDA function (Lovinger et al., 1989). Thus, it is possible that NTS1 agonists may also inhibit some effects of alcohol through a synergistic interaction with NMDA receptors.

### Neurotensin and alcohol in human studies

Two human studies have found links between *NTR1* single-nucleotide polymorphism (SNPs) and AUD in humans (Agrawal et al., 2011; Ma et al., 2013). Three *NTR1* SNPs (rs3787535, rs6089930, and rs3915568) were found to be significantly associated with consumption factor scores in 827 young adult twin women from the Missouri Adolescent Female Twin Registry (Agrawal et al., 2011). However, these findings were not replicated in a separate cohort. Another study observed significant associations between two *NTR1* SNPs (rs6011914 and rs2427422) and alcohol dependence in Han Chinese males (Ma et al., 2013). Thus, certain *NTR1* genotypes in humans may increase susceptibility for AUD.

Alcohol has also been shown to affect plasma NT levels in humans. Oral consumption of alcohol (1 g/kg) by healthy men transiently increased plasma NT levels (Szulc et al., 1991). Therefore, NT may be involved in the physiological effects of alcohol in humans.

### Neurotensin and alcohol in animal models

NT appears to contribute to the neurochemical and behavioral effects of alcohol in animal models. Administration of NT into the brain (since NT does not readily cross the BBB and is easily degraded) induces hypothermia (Bissette et al., 1976), hypolocomotion (Nemeroff et al., 1977), and striatal dopamine release (Kalivas and Duffy, 1990) similar to alcohol. NT also enhances alcohol-induced loss of righting reflex and hypothermia (Frye et al., 1981; Luttinger et al., 1983). Furthermore, cross-tolerance develops between alcohol and NT to their locomotor inhibitory and hypothermic effects, indicating that NT affects sensitivity to alcohol-induced intoxication (Erwin et al., 1995). LS and SS mice, which differ in their sensitivity to alcohol-induced sedation, were found to exhibit differences in brain NT concentration, brain NT receptor levels, and in behavioral responses to NT (Erwin and Jones, 1989; Erwin et al., 1990). Thus, differences in NT signaling in the brain may partially explain why LS mice are more sensitive to alcohol-induced sedation. In support of this, a couple of studies have identified quantitative trait loci (QTL) linked to both NTS1 levels in the striatum and sensitivity to alcohol hypnosis (Erwin and Jones, 1993; Erwin et al., 2001). NT has also been linked to alcohol consumption since P rats, which were selectively bred to consume high amounts of alcohol, were reported to have reduced NT levels in the frontal cortex relative to NP rats (Ehlers et al., 1999). Together, these findings suggest enhanced NT signaling increases sensitivity to the intoxicating effects of alcohol, which may reduce alcohol consumption.

Conversely, alcohol regulates NT signaling. Chronic alcohol exposure was reported to increase NT in the dorsal striatum in rats (Wachi et al., 1996). In mice, both chronic and acute alcohol decreased NTS1 and NTS2 in the NAc and midbrain (Campbell and Gene Erwin, 1993). These findings suggest that alcohol may increase NT levels and decrease NT receptor expression, which may reduce alcohol sensitivity and promote alcohol consumption. Alcohol-mediated reduction of NT receptors may explain why cross-tolerance exists between NT and alcohol. It also suggests that NT signaling contributes to tolerance to the intoxicating effects of alcohol.

#### Neurotensin receptor type 1 and alcohol

Since NTS1 inhibits D2R function and potentially regulates glutamate signaling, NTS1 is a promising therapeutic target for AUD. We have shown that NTS1 KO mice are less sensitive to the ataxic effect of alcohol and consume more alcohol relative to wildtype mice in a two-bottle choice drinking study (Table 1; Lee et al., 2010). In this same study, we used a BBB permeable synthetic NT analog, NT69L, to reduce two-bottle choice alcohol consumption while enhancing sensitivity to alcohol-mediated ataxia in mice. NT69L acts as an agonist with relatively equal affinity for NTS1 and NTS2 (Boules et al., 2006). However, it was observed that NT69L did not decrease alcohol consumption or enhance alcohol-induced ataxia in NTS1 KO mice (Lee et al., 2010). Our findings suggested that NTS1 activation increases sensitivity to alcohol intoxication while decreasing voluntary baseline alcohol consumption.

**Table 1:** **Alcohol consumption and alcohol reward behavior in gut-brain peptide mouse models**.

**Neuropeptide**	**Mouse**	**Alcohol consumption**	**Alcohol CPP**	**References**
Neurotensin	NTS1 KO	↑	ND	Lee et al., 2010
	NTS2 KO	↑	ND	Lee et al., 2011
	NT KO	ND	ND	
GLP-1	GLP-1 KO	ND	ND	
	GLP-1R KO	ND	ND	
Ghrelin	ghrelin KO	↓/–	↓	Jerlhag et al., 2011b; Bahi et al., 2013
	GHS-R1a KO	–	↓	Jerlhag et al., 2009
SP	NK1R KO	↓	↓	George et al., 2008; Thorsell et al., 2010
NPY	NPY KO	↑	ND	Thiele et al., 1998
	NPY OVREXP	↓	ND	Thiele et al., 1998
	Y1R KO	↑	ND	Thiele et al., 2002
	Y2R KO	↓	ND	Thiele et al., 2004
	Y5R KO	–	ND	Thiele et al., 2000

Abbreviations and symbols: ND, no data; ↓, decrease; ↑, increase; –, no change; OVREXP, overexpression.

NTS1 has also been implicated in sleep and psychiatric disorders which often co-occur with AUD and can increase the proclivity for relapse. NTS1 KO mice spent less time in rapid eye movement sleep (REM), but more time in non-REM sleep (Fitzpatrick et al., 2012). The same study found that NTS1 KO mice exhibited anxiety- and depression-like behaviors. Furthermore, there is a substantial amount of work demonstrating the antipsychotic effects of NTS1 agonists in rodents (Kinkead and Nemeroff, 2006; Boules et al., 2013). These data suggest that NTS1 agonists may also be useful in treating alcohol addiction with comorbid psychiatric or sleep disorders. However, additional research is needed to elucidate the precise role of NTS1 in alcohol dependence.

#### Neurotensin receptor type 2 and alcohol

NTS2 also seems to play a role in alcohol sensitivity and consumption. In LS and SS recombinant inbred strains, a negative correlation between NTS2 density in the frontal cortex and voluntary alcohol consumption was reported (Gehle and Erwin, 1998). More recently, we found that NTS2 KO mice were less sensitive to alcohol-induced sedation and consumed more alcohol in comparison to wildtype mice in a two-bottle choice paradigm (Table 1; Lee et al., 2011). However, NT69L attenuated baseline alcohol consumption and preference in wildtype as well as in NTS2 KO mice (Lee et al., 2011). This confirmed previous findings that NTS1 seems to be responsible for the effect of NT69L on reducing alcohol consumption. In future studies, the use of NTS2-selective agonists will help to elucidate the role of NTS2 in alcohol consumption. Although these studies suggest that NTS2 enhances sensitivity to higher hypnotic doses of alcohol while NTS1 increases sensitivity to lower ataxic doses of alcohol, and by these mechanisms both receptors may suppress alcohol consumption (Lee et al., 2011). Further research is necessary to determine if NTS2 may regulate alcohol consumption by other mechanisms besides simply increasing sensitivity to the sedative effect of alcohol.

### Summary

Recent animal behavioral data suggests that NTS1 agonists may be useful for suppressing baseline drinking or binge drinking (Lee et al., 2010). NT agonists could be a novel treatment for reducing alcohol consumption by enhancing the intoxicating effects and by possibly reducing the rewarding/reinforcing effects of alcohol. Interestingly, NT is thought to increase striatopallidal GABA transmission in the NAc, which may explain some of its effects (Li et al., 1995). Activation of striatopallidal neurons may reduce drug administration and decrease locomotor activity (Lobo and Nestler, 2011; Farrell et al., 2013). NT agonists may also reduce alcohol consumption by inhibiting alcohol-mediated glutamate signaling in the striatum or cortex (Li et al., 2011). Given the dopaminergic and glutamatergic neuromodulatory roles of NT in the striatum, further research is needed to uncover if NT agonists also affect alcohol reward, binge drinking, conditioned reinforcement and reinstatement in animal models.

## GLP-1

GLP-1 is a 30-a.a. peptide and a member of a class of molecules referred to as incretins, which facilitate food intake-stimulation of insulin release (Perfetti and Merkel, 2000). Clinical use of GLP-1 for diabetes was limited by its rapid degradation by dipeptidyl peptidase-4 (DPP-4) and other peptidases (Hansen et al., 1999). Then a major breakthrough occurred with the discovery of a peptide similar in structure and function to GLP-1, exendin-4 (Ex4), that is resistant to degradation by DPP-4 (Thorens et al., 1993; Young et al., 1999). Ex4 and another GLP-1 analog, liraglutide, are now FDA-approved for the treatment of type II diabetes. Besides glycemic control, there is increasing interest in GLP-1 for the treatment of obesity and addiction (Reddy et al., 2013; Skibicka, 2013). Central and peripheral administration of GLP-1 reduces food consumption, an effect that was thought to be primarily mediated by the hypothalamus and brainstem nuclei (Turton et al., 1996; Naslund et al., 1999; Schick et al., 2003; Hayes et al., 2009). GLP-1 is produced in the nucleus of the solitary tract in the brainstem, which projects to numerous areas of the CNS expressing the GLP-1 receptor (GLP-1R), such as the hypothalamus (Dickson et al., 2012). Since GLP-1 is rapidly degraded by DPP-4 and both peripheral and central GLP-1 reduces food consumption, it has been proposed that there may be a signaling link between peripheral GLP-1R activation and central GLP-1 release from the nucleus of the solitary tract (Trapp and Hisadome, 2011). One possible mechanism would be GLP-1R-mediated activation of the vagus nerve which projects to the nucleus of the solitary tract.

GLP-1R is widely expressed in brain, not just in the hypothalamus and brainstem. GLP-1R is detected in areas such as the hippocampus, amygdala, NAc, VTA, SN, and pituitary (Merchenthaler et al., 1999). Thus, the expression pattern of GLP-1R suggests GLP-1 has additional roles in the CNS. Recent work has implicated GLP-1 in learning, stress, and reward (During et al., 2003; Kinzig et al., 2003; Alhadeff et al., 2012). Therefore, central GLP-1 signaling could be a relevant therapeutic target for AUD.

### GLP-1 and corticostriatal-limbic circuitry

GLP-1 may decrease the rewarding effect of food and drugs of abuse by acting on the mesolimbic pathway (Dossat et al., 2011; Alhadeff et al., 2012; Dickson et al., 2012). Peripheral administration of the GLP-1 analog, Ex4, decreased food reward and food seeking in rats (Dickson et al., 2012). Microinjection of Ex4 into the VTA or into the NAc reduced motivation for food, as determined by progressive ratio responding for sucrose, and food consumption (Alhadeff et al., 2012; Dickson et al., 2012). These studies suggest that GLP-1R in the NAc and VTA may play a role in natural reward and reinforcement. Ex4 also reduced amphetamine- and cocaine-induced locomotion, reward and NAc dopamine release (Erreger et al., 2012; Graham et al., 2012; Egecioglu et al., 2013). However, Ex4 had no effect on accumbal dopamine levels alone (Egecioglu et al., 2013). These studies indicate that GLP-1R-mediated attenuation of reward may be linked to an inhibition of drug-induced dopamine release in the NAc.

In the mesolimbic system, neurons (such as those in the nucleus of the solitary tract) projecting to the VTA and NAc are the most likely source of endogenous GLP-1. However, the mechanism by which GLP-1 in the mesolimbic pathway reduces food or drug intake is not clear (Alhadeff et al., 2012). A recent study suggests that GLP-1R activation surprisingly increased dopaminergic neuron activity in the VTA (Mietlicki-Baase et al., 2013). Ex4 increased VTA tyrosine hydroxylase expression which suggests that GLP-1R activation may increase dopamine synthesis and release. In this same study, the anorexic effect of microinjection of Ex4 into the VTA was reduced by an AMPA/kainate antagonist and Ex4 increased AMPA/kainate receptor-mediated sEPSC frequency in VTA dopaminergic neurons. Thus, these findings suggest that presynaptic GLP-1R activation on glutamatergic terminals in the VTA may increase VTA dopaminergic neuron firing through AMPA/kainate neurotransmission. Since increased VTA firing would be expected to increase food intake and reward sensitivity, it is controversial. One possibility, as suggested by a recent study, is that GLP-1R activation in the VTA may preferentially increase dopamine in the amygdala rather than in the NAc (Anderberg et al., 2014). Amygdala dopamine D2R activation was observed to decrease food intake and *i.c.v.* administration of Ex4 increased dopamine turnover in the amygdala (Anderberg et al., 2014). The anorexigenic effect of Ex4 appeared to be at least partially mediated by D2R activation in the amygdala since a D2R antagonist attenuated the effect of Ex4 on suppressing food intake. Additional studies are required to clarify whether GLP-1R activation in the VTA increases amygdala dopamine levels as we propose in Figure 3A. Regardless, this does not explain how microinjection of Ex4 in the NAc reduces food intake.

To address the role of GLP-1 in the NAc, the same group investigated how Ex4 affects the activity of NAc MSNs (Mietlicki-Baase et al., 2014). Interestingly, Mietlicki-Baase et al. (2014) found, similar to the VTA, that Ex4 increases the activity of NAc MSNs by enhancing AMPA/kainate receptor-mediated mEPSCs and this effect appeared to be independent of postsynaptic GLP-1Rs on MSNs. Secondly, Ex4 in the NAc reduced food intake and this effect was reduced by an AMPA/kainate receptor antagonist. Finally, Ex4 alone did not enhance NAc dopamine as measured by *ex vivo* fast-scan cyclic voltammetry. Together, this suggests that Ex4 acts GLP-1Rs on presynaptic glutamatergic terminals to excite MSNs and reduce food intake. As shown in Figure 3B, since it is hypothesized that activation of striatopallidal neurons reduces administration of drugs of abuse, it is possible that GLP-1 may predominantly excite MSNs in the indirect pathway by this glutamatergic mechanism (Lobo and Nestler, 2011). Thus, GLP-1-mediated activation of MSNs may reduce the administration of rewarding substances, such as alcohol, by increasing the inhibition of downstream targets of the NAc.

### GLP-1 and alcohol in humans

GLP-1 analogs have been deemed clinically safe, but there is no clear evidence that they reduce alcohol intake in humans. However, some studies have shown that bariatric surgery reduces alcohol intake in humans while increasing circulating GLP-1 in response to food (Woodard et al., 2011; Davis et al., 2012; Yousseif et al., 2014). Increased circulating GLP-1 is thought to play a key role in the efficacy of bariatric surgery. These studies imply that GLP-1 analogs may be beneficial in reducing alcohol intake in humans.

### GLP-1 and alcohol in animal models

GLP-1R activation has been shown to reduce alcohol consumption, alcohol reward, and motivated alcohol seeking. GLP-1R activation inhibited alcohol reward as demonstrated by reduced alcohol-induced locomotor activity, CPP and accumbal dopamine release with peripheral Ex4 administration in outbred mice (Egecioglu et al., 2012). Ex4 also reduced alcohol intake in an intermittent alcohol access paradigm and the break point for progressive ratio operant self-administration of alcohol in Wistar rats (Egecioglu et al., 2012). This implies that GLP-1R activation suppresses motivation to obtain alcohol. Corroborating these findings, another study reported that peripheral administration of GLP-1 and/or Ex4 reduced alcohol consumption in an intermittent access paradigm in Wistar rats and alcohol CPP in outbred mice (Shirazi et al., 2013). Moreover, a peripherally administered GLP-1R antagonist increased alcohol consumption while GLP-1R activation in the VTA was sufficient to reduce alcohol consumption in rats, indicating that the effect may be mediated by GLP-1R in the VTA (Shirazi et al., 2013). The study also indicated that within a certain dosage range, GLP-1R agonists may reduce alcohol consumption without affecting preference for natural rewards, since a lower dose of Ex4 that reduced alcohol intake did not alter water or food consumption (Shirazi et al., 2013).

Ex4 also appears to more effectively reduce alcohol consumption in rats consuming higher amounts of alcohol, suggesting that GLP-1 analogs may treat heavy drinking or binge drinking (Shirazi et al., 2013). Chronic heavy drinking may lead to brain neurodegeneration associated with memory and motor deficits. Approximately 10% of dementia cases have been linked to alcohol-related dementia (Gupta and Warner, 2008). Interestingly, Ex4 promoted adult neurogenesis *in vitro* and *in vivo* (Bertilsson et al., 2008), indicating that GLP-1R agonists could slow or even reverse neurodegenerative processes (Perry et al., 2002). Ex4 also improved memory performance and reduced depression-like behavior (Isacson et al., 2011). The potential cognitive and neurogenesis enhancing effects of GLP-analogs may be an additional benefit for using GLP-1R agonists as a treatment for AUD.

### Summary

GLP-1 analogs, such as Ex4, may be effective therapeutics for AUD. Preclinical data suggest that Ex4 may reduce alcohol reward and motivation. Furthermore, Ex4 may be especially useful for suppressing heavy drinking and alcohol-mediated neurodegeneration. However, more work is necessary to uncover the mechanism behind the anti-drinking effect of GLP-1 analogs. The majority of studies have focused on the effect of GLP-1R activation on the mesolimbic pathway. Recent findings indicate that GLP-1R activation may reduce the administration of rewarding substances by promoting glutamatergic signaling in the VTA and NAc, but further studies are needed to confirm this theory and clarify whether mesolimbic dopaminergic signaling is important. GLP-1R is also found in the SN and it is possible that the nigrostriatal pathway could play an important role in the suppressive action on Ex4 on alcohol consumption (Merchenthaler et al., 1999).

## Ghrelin

Ghrelin is a 28-a.a. peptide that is mainly synthesized and released by mucosal cells in the stomach. Ghrelin was originally named after its ability to act on the growth hormone secretagogue receptor 1a (GHS-R1a) in the pituitary to stimulate growth hormone release (Kojima et al., 1999). Acylation of ghrelin, which is catalyzed by the enzyme ghrelin O-acyltransferase (GOAT), is required for ghrelin to bind to GHS-R1a (Yang et al., 2008).

Ghrelin is well known for its orexigenic effects. Ghrelin level in the blood is higher after fasting and lower after food intake (Cummings et al., 2001). Both peripheral and central administration of ghrelin stimulates food intake and increases body weight (Tschöp et al., 2000; Wren et al., 2000). GHS-R1a blockade has the opposite effect of reducing food consumption (Salome et al., 2009). Ghrelin primarily promotes food consumption by acting indirectly and directly on the hypothalamus. Ghrelin indirectly activates the hypothalamus through the vagus nerve which acts on the nucleus of the solitary tract in the brainstem which then projects to the arcuate nucleus of the hypothalamus (Date et al., 2002). Ghrelin may also directly act on homeostatic feeding areas in the brain since ghrelin crosses the BBB (Banks et al., 2002); it can also reach the hypothalamus through areas which are not fully protected by the BBB (Merchenthaler, 1991). In support of this, central administration of ghrelin reduces feeding and a study has shown that peripheral ghrelin still increases feeding after vagal deafferentation (Tschöp et al., 2000; Arnold et al., 2006).

Although most circulating ghrelin comes from the stomach, ghrelin mRNA is widely detected in the brain; however, it is debatable whether ghrelin is released at physiologically meaningful levels in the CNS (Gnanapavan et al., 2002; Furness et al., 2011). Nevertheless, since peripheral ghrelin can cross the BBB (Banks et al., 2002), and GHS-R1a possesses constitutive (or ligand-independent) activity (Holst et al., 2003), GHS-R1a likely exerts its effect on brain function even with inadequate production of central ghrelin. In the brain, GHS-R1a is also expressed in areas outside the hypothalamus, such as in the hippocampus, SN, VTA, cortex and amygdala (Guan et al., 1997; Zigman et al., 2006; Alvarez-Crespo et al., 2012). Recently ghrelin was shown to affect reward (Skibicka and Dickson, 2011), locomotion (Jerlhag et al., 2006), mood (Kanoski et al., 2013), and memory (Alvarez-Crespo et al., 2012).

### Ghrelin and corticostriatal-limbic circuitry

Ghrelin and GHS-R1a have been shown to affect mesocorticolimbic dopaminergic signaling. When administered intravenously to humans, functional magnetic resonance imaging (fMRI) revealed ghrelin increased the activity of the VTA and the regions it projects to (amygdala, striatum, hippocampus, cortex) when viewing pictures of food (Malik et al., 2008). GHS-R1a is expressed presynaptically and postsynaptically in the VTA (Abizaid et al., 2006) as well as on cholinergic neurons in the laterodorsal tegmental area (LDTg) (Dickson et al., 2010). Cholinergic neurons in the LDTg project to and excite VTA dopaminergic neurons (Woolf, 1991). In mice, microinjection of ghrelin into the VTA or LDTg increased dopamine in the NAc (Jerlhag et al., 2007). This suggests that ghrelin may act locally on VTA neurons or increase the activity of mesolimbic dopaminergic neurons through indirect mechanisms, such as enhancing cholinergic input from the LDTg (Figure 4A). In fact, a nicotinic acetylcholine receptor antagonist was shown to block the effect of ghrelin in the LDTg on dopamine release in the NAc (Jerlhag et al., 2008). This suggests that nicotinic acetylcholine receptors can modulate the effects of ghrelin on mesolimbic dopaminergic signaling and reward. It is possible that peripheral ghrelin regulates mesolimbic circuitry since peripherally administered ghrelin increased NAc dopamine whereas GHS-R1a antagonist administration had the opposite effect (Jerlhag, 2008; Jerlhag et al., 2010). Ghrelin did not increase the activity of VTA dopaminergic neurons in mice lacking GHS-R1a, suggesting that GHS-R1a-mediated signaling plays an essential role in regulating dopaminergic neuron activity (Abizaid et al., 2006). Ghrelin-mediated excitation of VTA dopaminergic neurons may be partially attributed to its effects on synaptic plasticity. Ghrelin administration increased excitatory and decreased inhibitory synapses onto VTA dopaminergic neurons in mice (Abizaid et al., 2006). Chronic ghrelin administration altered the expression of dopaminergic and nicotinic acetylcholine receptors in the mesolimbic reward circuitry (Skibicka et al., 2012). These results suggest that changes in ghrelin signaling, such as those mediated by alcohol, could lead to long-lasting adaptations in the synaptic plasticity of the reward system.

The actions of ghrelin on mesoaccumbal dopamine appear to mediate its effects on reward sensitivity. Microinjection of ghrelin into the VTA and NAc increased feeding in rodents (Naleid et al., 2005; Abizaid et al., 2006), while lesioning the VTA blocked ghrelin-induced increases in palatable food consumption (Egecioglu et al., 2010). Ghrelin-mediated increase in natural reward consumption is dependent on GHS-R1a, since antagonism or genetic deletion of GHS-R1a in rodents reduced palatable food consumption, but not standard chow intake (Egecioglu et al., 2010). Furthermore, palatable food-mediated CPP was increased by ghrelin, but blocked by a GHS-R1a antagonist or genetic deletion of GHS-R1a or ghrelin (Egecioglu et al., 2010; Perello et al., 2010; Disse et al., 2011). Ghrelin-mediated increase in NAc dopamine signaling has been shown to play a crucial role in reward sensitivity since dopamine D1R and D2R antagonists in the NAc attenuate the ability of VTA ghrelin to increase operant responding for sucrose (Skibicka et al., 2013). These studies indicate that ghrelin increases reward sensitivity, which is most likely through its effects on VTA dopaminergic neurons. The effect of ghrelin on dopaminergic VTA neuronal activity appears to be modulated by glutamatergic signaling. A NMDA receptor antagonist blocked the effects of VTA ghrelin on dopamine release in the NAc and on locomotor stimulation (Jerlhag et al., 2011a). This suggests that glutamatergic projections to the VTA are modulated by the actions of ghrelin (Figure 4B).

Microinjection of ghrelin into the ventral, but not the dorsal hippocampus has been shown to increase chow intake in Sprague–Dawley rats (Kanoski et al., 2013). Ventral hippocampus ghrelin also increased the motivation for natural reward as determined by an increased breakpoint under a progressive ratio schedule for sucrose. Furthermore, ghrelin in the ventral hippocampus increased cue-induced feeding. As shown in Figure 4C, these effects may be mediated by increased glutamatergic signaling from the ventral hippocampus to the NAc since microinjection of ghrelin into the ventral hippocampus increased phosphorylated tyrosine hydroxylase in the NAc, suggesting increased dopamine release (Kanoski et al., 2013). The ventral hippocampus has strong glutamatergic projections to the NAc and may directly or indirectly affect the local release of dopamine in the NAc from presynaptic VTA terminals (Groenewegen et al., 1996; McDonald, 1998). Inactivation of the ventral hippocampus attenuated cue and cocaine-induced reinstatement (Rogers and See, 2007). Other studies have shown that drug cues activate the hippocampus (Topple et al., 1998; Kufahl et al., 2009). It is possible that the ventral hippocampus is essential in retrieval of alcohol-cue associations. Therefore, ghrelin in the ventral hippocampus may increase the recall of cues associated with alcohol, which could promote relapse. Microinjection of ghrelin into the amygdala also increased chow intake (Alvarez-Crespo et al., 2012). The amygdala plays a key role in assigning emotional and motivational value to contextual cues. Therefore, ghrelin in the hippocampus or amygdala may regulate alcohol reinstatement.

### Ghrelin and alcohol in humans

Since alcohol consumption and food seeking are regulated by overlapping neuronal mechanisms, studies sought to determine if alcohol use in humans would affect circulating ghrelin. Several studies found that an acute low (0.55–0.6 g/kg) dose of oral alcohol in humans decreased total and acyl-ghrelin levels (Calissendorff et al., 2005, 2006; Zimmermann et al., 2007). In support of these findings, active drinking AUD patients exhibited lower plasma ghrelin levels compared with controls (Addolorato et al., 2006; Badaoui et al., 2008). More recently, *i.v.* administration of alcohol was reported to reduce fasting-mediated ghrelin levels in social drinkers (Leggio et al., 2013). Since oral alcohol administration greatly decreased circulating ghrelin where *i.v.* administration only blunted the fasting-induced ghrelin, the local effect of alcohol on endocrine cells in the stomach may be an important factor in alcohol-mediated reduction of peripheral ghrelin levels. Evidence suggests that alcohol may decrease circulating ghrelin beyond a level that would be expected by the caloric value of alcohol (Landgren et al., 2008). Furthermore, alcohol may more specifically affect ghrelin, but not other gut peptides like GLP-1, insulin and PPY (Calissendorff et al., 2005, 2006; Zimmermann et al., 2007; Leggio et al., 2013).

Alcohol withdrawal appears to have the opposite effect and increase ghrelin levels. An early study compared acyl-ghrelin plasma levels in alcohol dependents that were abstinent for at least 30 days, with levels in healthy controls. Abstinent individuals exhibited higher levels of plasma ghrelin that were positively correlated with the duration of abstinence (Kim et al., 2005). In another study, higher total plasma ghrelin levels were found after early abstinence (at least 72 h) (Kraus et al., 2005). Another study reported the opposite finding where baseline serum total ghrelin levels were decreased in early abstinent individuals (72 h) relative to non-abstinent alcohol dependents (Leggio et al., 2012). However, this may be because Kraus et al. (2005) measured ghrelin levels following 72 h or more after abstinence. It is possible that plasma ghrelin levels are not clearly elevated until more than 72 h after abstinence (Kraus et al., 2005). However as previously observed, Leggio et al. (2012) found ghrelin levels were elevated in abstinent individuals over time. Another study has also supported these findings by demonstrating that plasma acyl-ghrelin levels slightly increased during abstinence (0–14 days) (Koopmann et al., 2012). However, there are studies that contradict these findings by showing decreased total ghrelin levels in abstinent alcohol dependents (Wurst et al., 2007; de Timary et al., 2012). These conflicting results may be due to differences in looking at active acyl-ghrelin vs. total ghrelin, gender differences, sample sizes and the time after abstinence. Together most of these data point to a link between early alcohol withdrawal and increasing circulating ghrelin levels. A better understanding of the factors that contribute to ghrelin levels in active drinkers could potentially lead to using plasma ghrelin as a biomarker for AUD.

There are also a number studies supporting a role for ghrelin in alcohol craving in humans (Addolorato et al., 2006; Koopmann et al., 2012; Leggio et al., 2012). Total plasma ghrelin levels were positively correlated with craving as determined by the Obsessive Compulsive Drinking Scale (OCDS) in actively drinking alcohol dependents (Addolorato et al., 2006). In another study, total plasma ghrelin levels were quantified in alcohol dependents at several time points after abstinence and compared to their craving scores as determined by the Pennsylvania Alcohol Craving Scores (PACS) and OCDS (Leggio et al., 2012). Total plasma ghrelin levels quantified 72 h after abstinence were positively correlated with PACS scores acquired after 2 weeks of abstinence, and PACS and OCDS scores acquired after 6 and 12 weeks of abstinence. Another study showed that plasma acyl-ghrelin levels were positively correlated with OCDS scores (Koopmann et al., 2012). The majority of studies suggest that reducing ghrelin levels or blocking the ghrelin receptor may reduce alcohol craving.

Lastly, there have been some genetic studies implicating ghrelin in alcohol dependence. One study found a SNP (rs2232165) in exon 1 of the *GHS-R1A* gene to be linked with heavy alcohol use (Landgren et al., 2008). However, the same group was not able to replicate an association between the same SNP and alcohol use in a population of Caucasian females with alcohol dependence (Landgren et al., 2010). On the other hand, they did find weak associations between a haplotype of the pro-ghrelin gene *GHRL*, and self-reported greater alcohol withdrawal symptoms and paternal alcohol dependence (Landgren et al., 2010). There was also an association between a haplotype for *GHS-R1A* and early-onset alcohol dependence. These studies indicate that disruption of the ghrelin system may predispose some individuals to AUD. Future studies will elucidate how these haplotypes and/or SNP are linked with AUD.

### Ghrelin and alcohol in animal models

Preclinical studies have shown both normal baseline and decreased alcohol consumption by ghrelin KO mice relative to wildtype mice (Table 1; Jerlhag et al., 2011b; Bahi et al., 2013). GHS-R1a KO mice also consume similar levels of alcohol relative to wildtype mice in a limited access paradigm (Table 1; Jerlhag et al., 2009). Compensatory effects may explain why these mice show no difference in alcohol consumption. Other studies have demonstrated correlations between ghrelin genes and/or levels and alcohol-related behaviors in rodents. High alcohol preferring AA rats exhibited increased GHS-R1a gene expression in the NAc, VTA, amygdala, hippocampus and PFC after alcohol consumption relative to non-alcohol preferring ANA rats (Landgren et al., 2011). However, another study showed that 10 months of alcohol consumption decreased GHS-R1a expression in the VTA of rats (Suchankova et al., 2013). P rats and WHP rats showed greater reductions in plasma acyl-ghrelin levels after alcohol consumption relative to NP and WLP rats (Szulc et al., 2013). Together these studies imply that alcohol affects circulating ghrelin and central ghrelin signaling. It is possible that in the early stages of alcohol drinking ghrelin levels may be reduced and GHS-R1a expression may be increased. Longer periods of alcohol consumption and withdrawal may lead to increased ghrelin and decreased GHS-R1a expression. Future work using transgenic mice, viral-mediated gene knockdown and different drinking paradigms will be important to clarify the relationship between ghrelin signaling in the brain and alcohol consumption. Also, since ghrelin is expressed in the CNS, it is possible that alcohol could have a differential effect on ghrelin levels in the corticostriatal-limbic circuitry relative to the peripheral plasma levels.

Pharmacological studies using animal models demonstrate the importance of ghrelin in AUD. Alcohol intake in a limited access paradigm was increased by ghrelin administered *i.c.v* to C57BL/6J mice (Jerlhag et al., 2009). GHS-R1a antagonists have been shown to suppress alcohol consumption using several different paradigms (two-bottle choice, limited access, intermittent access and fixed ratio operant self-administration) (Jerlhag et al., 2009; Landgren et al., 2012; Bahi et al., 2013; Suchankova et al., 2013). In terms of the brain regions involved, ghrelin increased alcohol consumption in a limited access paradigm when administered into the VTA and into the LDTg (Jerlhag et al., 2009). Thus, ghrelin may increase alcohol consumption by enhancing mesolimbic dopaminergic signaling. In support of a role for ghrelin in reward circuitry, genetic deletion of ghrelin, GHS-R1a, or treatment with a GHS-R1a antagonist blocked alcohol-induced CPP (Table 1; Jerlhag et al., 2009; Bahi et al., 2013). Furthermore, alcohol-induced dopamine release in the NAc was reduced by GHS-R1a antagonists and blocked in GHS-R1a KO mice and ghrelin KO mice (Jerlhag et al., 2009, 2011b). These studies suggest that ghrelin is necessary for alcohol-induced dopamine release in the NAc and alcohol reward, which likely contributes to ghrelin's effects on alcohol consumption. However, it is still not clear if acute alcohol increases ghrelin in the VTA and if the mechanism is local or through regulation of circulating ghrelin.

Enhanced alcohol reward sensitivity increases the risk for binge drinking (King et al., 2011). Interestingly, a GHS-R1a antagonist D-Lys3-GHRP-6 reduced mouse binge-like alcohol drinking-in-the-dark (Kaur and Ryabinin, 2010). However, it has also been reported that peripheral administration of a dose of ghrelin that increases food consumption did not increase drinking-in-the-dark (Lyons et al., 2008). A recent study, suggests that the discrepancy may be because circulating acylated ghrelin does not play a significant role in alcohol consumption and reward (Jerlhag et al., 2014). In this study, mice were administered NOX-B11-2 which binds to and neutralizes acyl-ghrelin only in the periphery, since it cannot cross the BBB. NOX-B11-2 had no effect on alcohol-induced dopamine release, alcohol consumption or alcohol-mediated CPP which implies that peripheral ghrelin does not play an important role in alcohol-related behaviors. NOX-B11-2 did reduce food consumption which supports previous findings that peripheral ghrelin regulates food intake. Thus, more work is needed to determine if peripheral or central ghrelin plays a greater role in regulating alcohol consumption.

GHS-R1a antagonists have been demonstrated to attenuate alcohol consumption, reward and reinforcement, but to be an effective therapeutic for alcohol dependence it should prevent relapse. A recent study implies that the GHS-R1a antagonist JMV2959 may be useful in preventing relapse (Suchankova et al., 2013). JMV2959 prevented the alcohol deprivation-mediated increase in alcohol consumption in rats. It was also shown that JMV2959 suppressed alcohol consumption of rats that had been drinking for 8 months; tolerance did not develop to the effect and alcohol consumption did not increase after treatment stopped (Suchankova et al., 2013). This work, along with other preclinical data, demonstrates that GHS-R1a is a promising target for the treatment of alcohol dependence. Alternatively, GOAT inhibitors could also be a potential therapeutic target since the inhibition of GOAT is expected to reduce circulating acyl-ghrelin levels.

### Summary

The majority of the research on ghrelin in AUD has focused on its effects on the mesolimbic pathway and alcohol reward. GHS-R1a antagonists appear to reduce baseline alcohol consumption by suppressing alcohol-mediated dopamine release in the NAc and thus alcohol reward. GHS-R1a antagonists also inhibit operant responding for alcohol, implying that GHS-R1a antagonists suppress positive reinforcement for alcohol. More recently, preclinical work has suggested that GHS-R1a antagonists may be useful for reducing relapse and craving in AUD. The GHS-R1a antagonist JMV2959 was shown to reduce alcohol deprivation-mediated increase in alcohol consumption (Suchankova et al., 2013). In support of this finding, human studies have demonstrated positive correlations between plasma ghrelin levels and craving scores. Therefore, GHS-R1a antagonists could potentially be used to suppress alcohol craving and relapse. Within corticostriatal-limbic circuitry, the ventral hippocampus and amygdala were identified as key sites where ghrelin affects food intake (Alvarez-Crespo et al., 2012; Kanoski et al., 2013). These two brain regions are thought to play an important role in conditioned reinforcement and relapse in AUD. In future work, it would be interesting to investigate if GHS-R1a antagonists may suppress craving and prevent relapse by its actions in the ventral hippocampus or the amygdala.

## Substance P

Substance P (SP) is an 11-a.a. peptide and a member of the tachykinin family. SP was originally isolated from the horse intestine and brain (Us and Gaddum, 1931). Since its discovery, SP has been identified in a wide range of tissues in the periphery and CNS. In the CNS, SP is found in regions such as the cortex, amygdala, hippocampus, hypothalamus, NAc, caudate, putamen, dorsal raphe, locus coeruleus, and SN (Shults et al., 1984). In agreement with the expression profile of SP, it has been implicated in mood (Santarelli et al., 2001), stress (Papp et al., 2000), reward sensitivity (Thorsell et al., 2010), energy homeostasis (Karagiannides et al., 2008), pain (Hill, 2000), and learning (Huston and Hasenohrl, 1995).

There are three tachykinin receptor subtypes, neurokinin 1 receptor (NK1R), neurokinin 2 receptor (NK2R), and neurokinin 3 receptor (NK3R) (Severini et al., 2002). All three receptors are G-protein-coupled receptors. SP preferentially binds to NK1R and NK1R is expressed in many of the same brain regions as SP (Mantyh et al., 1984; Nakaya et al., 1994). NK1R and SP have been increasingly implicated in affective disorders. NK1R antagonists or genetic deletion of NK1R induces anxiolytic and antidepressant-like effects in animal models (Teixeira et al., 1996; File, 1997; Papp et al., 2000; Rupniak et al., 2000; Santarelli et al., 2001). There is also accumulating evidence for a role of SP/NK1R in food intake and addiction (Murtra et al., 2000; Karagiannides et al., 2008; Ramalho et al., 2013). A recent series of studies suggest that NK1R antagonists have potential to be a novel therapeutic for AUD (George et al., 2008; Thorsell et al., 2010; Schank et al., 2011, 2014).

### Substance P and corticostriatal-limbic circuitry

Inhibition or genetic deletion of NK1R has been shown to reduce intake of natural reward and drugs of abuse (Ripley et al., 2002; Karagiannides et al., 2008; Steensland et al., 2010; Thorsell et al., 2010; Schank et al., 2013). One possible mechanism by which NK1R inhibition may inhibit the administration of rewarding substances is through modulation of mesolimbic dopaminergic signaling. SP is found presynaptically (Tamiya et al., 1990) and NK1R is expressed postsynaptically on dopaminergic neurons and other neuron subtypes in the VTA (Lessard and Pickel, 2005). Peripheral administration of SP increased dopamine in the NAc (Boix et al., 1992) and microinjection of SP analogs into the VTA indicate that this effect is mediated by NK1R activation in the VTA (Figure 5A) (Elliott et al., 1986, 1991; Barnes et al., 1990). Conversely, an NK1R antagonist was shown to reduce the number of spontaneously active dopaminergic neurons in the VTA, further supporting that NK1R antagonists may reduce the intake of rewarding substances by suppressing activation of the mesolimbic pathway (Minabe et al., 1996). SP or an SP analog in the VTA has also increased dopamine or its metabolites in other postsynaptic targets (e.g., PFC, amygdala) (Deutch et al., 1985; Elliott et al., 1986; Barnes et al., 1990). Furthermore, an NK1R antagonist reduced the number of active dopaminergic neurons in the SN, which indicates that NK1R inhibition may also suppress the nigrostriatal pathway (Minabe et al., 1996). Therefore, NK1R modulation of dopamine signaling in corticostriatal-limbic circuitry could mechanistically explain the effects of the SP/NK1R system on behaviors related to AUD.

The ventral pallidum is another important brain region for SP/NK1R signaling. Microinjection of SP into the ventral pallidum induces CPP and this effect is inhibited by a NK1R antagonist (Nikolaus et al., 1999). The ventral pallidum receives input from the cortex, amygdala, NAc, and other areas that express SP (Reep and Winans, 1982; Napier et al., 1995; Mitrovic and Napier, 1998). An analog of SP increased the excitability of the ventral pallidum which is interesting since inactivation of the ventral pallidum is thought to prevent drug- or stress-induced reinstatement (Napier et al., 1995; McFarland and Kalivas, 2001; McFarland et al., 2004). Therefore, NK1R antagonists may reduce addictive behaviors through suppressing activity of the ventral pallidum (Figure 5B).

SP may also regulate addictive behaviors through affecting neurotransmission in the amygdala. The rewarding effects of morphine were suppressed by lesioning NK1R-expressing cells in the amygdala (Gadd et al., 2003). Besides regulating the rewarding effects of substances, inhibition of NK1R in the amygdala may reduce stress-induced relapse or negative affect associated with AUD. Microinjection of a SP receptor antagonist into the amygdala was anxiolytic, where SP was anxiogenic (Ebner et al., 2004; Zhao et al., 2009). Furthermore, emotional stressors increased SP in the amygdala (Ebner et al., 2004). Thus, inhibiting amygdala SP signaling may reduce the effects of stress and anxiety on promoting addictive behaviors (Figure 5B). Actually, a NK1R antagonist was shown to suppress stress-induced reinstatement for cocaine and we will discuss below how NK1R antagonists are promising therapeutics for the prevention of stress-induced relapse in AUD (Schank et al., 2014).

### Substance P and alcohol in humans

The importance of SP/NK1R system in AUD is supported by the observation of significant associations between the NK1R gene (*TACR1*; rs6715729 and two haplotypes) and alcohol dependence (Seneviratne et al., 2009). *TACR1* SNPs (rs3771863, rs3755459, and rs1106855) were also associated with blood oxygen level dependent (BOLD) responses to the taste of alcohol in the medial PFC, putamen and insula, as measured by fMRI in heavy drinkers (Blaine et al., 2013). This implies that the SP/NK1R system may regulate alcohol-related behaviors by acting on circuitry in the cortex and dorsal striatum in humans. The same study also reported associations between *TACR1* SNPs (rs10490308, rs11688000, rs3771863) and symptoms of alcohol dependence.

Clinical studies suggest that NK1R antagonists may be useful for suppressing stress-induced craving for alcohol. A randomized controlled study observed that detoxified patients with AUD who were given NK1R antagonist, LY686017, showed improved craving control, wellbeing, and stress response after social stress test or alcohol-cue challenge compared to placebo counterparts (George et al., 2008). By measuring BOLD activity by fMRI, LY686017 was found to suppress increased reactivity to negative emotional stimuli and enhance responses to positive emotional stimuli that were blunted in alcohol dependents (George et al., 2008). In addition, LY686017 reduced activation of the insula, which is involved in craving and addictive behavior (George et al., 2008; Naqvi and Bechara, 2009). The fact that LY686017 reduced stress-related craving and normalized responses to emotional stimuli, suggests the effect of NK1R antagonists on stress-related circuitry may be an important mechanism behind their therapeutic effect. Another clinical study examining the efficacy of NK1R antagonist, aprepitant, as a treatment for PTSD comorbid with alcohol dependence did not observe any treatment effect on PTSD symptoms and subjective and physiological responses to stress or alcohol cues, but fMRI showed a robust potentiation in the ventromedial PFC, suggesting that NK1R antagonism may be beneficial for alcoholic patients with PTSD to some extent (Kwako et al., 2014).

### Substance P and alcohol in animal models

Animal studies point to an association between disruption of SP/NK1R signaling and increased risk for alcohol-related problems. For example, SP immunoreactivity or expression is reduced in the frontal cortex and central nucleus of the amygdala (CeA) of alcohol-naïve P rats relative to NP rats (Slawecki et al., 2001; Yang et al., 2009). As a possible compensatory effect, P rats exhibited elevated expression of NK1R and receptor binding in the CeA (Schank et al., 2013). The *TACR1* SNP, -1372G/C, was found to affect TACR1 expression, where presence of the C-allele was associated with increased *TACR1* expression (Schank et al., 2013). All P rats, compared to only 18% of the Wistar rats, were homozygous for the C-allele, which may explain the increased amygdala NK1R expression in P rats (Schank et al., 2013). These results suggest that dysregulated SP/NK1R signaling may increase the risk for developing AUD. Conversely, alcohol may disrupt SP signaling, which could facilitate the development of AUD since chronic alcohol exposure increased SP immunoreactivity in the caudate and decreased SP immunoreactivity in the frontal cortex of rats (Slawecki et al., 2005).

Preclinical studies have demonstrated that disruption of SP/NK1R signaling alters alcohol-related behaviors in rodents. Genetic deletion of NK1R in mice reduced two-bottle choice alcohol consumption, increased alcohol-mediated sedation, and attenuated alcohol reward relative to wildtype mice (Table 1; George et al., 2008; Thorsell et al., 2010). Furthermore, NK1R KO mice showed reduced alcohol consumption in an escalated drinking model which involved intermittent access to 15% alcohol (Thorsell et al., 2010). NK1R antagonists had a similar effect and reduced two-bottle choice drinking and operant self-administration of alcohol in mice and rats (Steensland et al., 2010; Thorsell et al., 2010; Schank et al., 2013). However, the NK1R antagonist ezlopitant had no effect on the breakpoint for alcohol in a progressive ratio operant responding schedule and more effectively reduced sucrose self-administration in Long Evans rats (Steensland et al., 2010). This suggests that ezlopitant non-specifically reduces reward sensitivity at the dose required to decrease responding for alcohol consumption and does not potently suppress motivation to seek alcohol. However, another study showed that the NK1R antagonist L822429 reduced progressive ratio responding for alcohol by P rats, but not by Wistar rats (Schank et al., 2013). L822429 did not reduce saccharine intake like ezlopitant (Schank et al., 2013). Thus, it is possible that NK1R antagonists may be specifically beneficial for attenuating alcohol seeking in those with a disrupted SP/NK1R system. Also, P rats have been shown to exhibit an anxiety-like phenotype and may be more sensitive to the effect of NK1R antagonists on reducing negative reinforcement for alcohol (Stewart et al., 1993).

There is some evidence that the amygdala may be a target for the therapeutic effect of NK1R antagonists. Intra-amygdala infusion of the NK1R antagonist L822429 reduced alcohol self-administration in P rats while injections in the PFC did not (Schank et al., 2013). The amygdala is a component of the extended stress system, thus may play an important role in motivating drug seeking (reviewed by Schank, 2014). In fact, some preclinical studies indicate that NK1R antagonists may suppress stress-mediated alcohol relapse. The NK1R antagonist L822429 blocked footshock stress-induced reinstatement for alcohol seeking in Long Evans rats (Schank et al., 2011). L822429 also suppressed yohimbine (pharmacological stressor)-induced reinstatement of alcohol seeking at a dose that did not affect alcohol or saccharine administration, suggesting that stress-induced alcohol reinstatement is especially sensitive to NK1R antagonists (Schank et al., 2014). It is possible that NK1R antagonists reduce alcohol intake by multiple mechanisms, which involve both positive reinforcement and negative reinforcement for alcohol.

### Summary

Preclinical studies have demonstrated SP/NK1R system's involvement in regulating voluntary alcohol administration, but more studies on the SP/NK1R signaling among corticostriatal-limbic structures is still needed to ascertain the areas of action and their interconnections for the specific effects elicited, such as stress relief and reduced motivation to seek alcohol. Both preclinical and clinical studies support that NK1R antagonism could be a potential treatment for reducing alcohol consumption, possibly through craving reduction and stress response alleviation. However, there is still a lack of clinical evidence for NK1R antagonist efficacy in reducing alcohol consumption or improving abstinence length. Future studies need to be conducted to confirm the usefulness of these antagonists in abstinence maintenance and to determine the specific alcohol dependent patients who will benefit most from them such as those with comorbid PTSD.

## NPY

NPY is a 36-a.a. neurotransmitter that is widely distributed in both the central and peripheral nervous system (Tatemoto et al., 1982; Gray and Morley, 1986). Thus, NPY influences a wide range of physiologic functions. NPY is best known for its orexigenic and anxiolytic properties (Badia-Elder et al., 2007). There are five G-protein-coupled NPY receptor subtypes: Y1R, Y2R, Y4R, Y5R, and Y6R (Blomqvist and Herzog, 1997). Y1R is mainly expressed postsynaptically and is activated by full-length NPY (Sheikh et al., 1989; Widdowson, 1993; Gehlert and Gackenheimer, 1997). In the CNS, Y1R is expressed in several brain regions including the cerebral cortex, striatum, amygdala, hypothalamus, thalamus, hippocampus and the nucleus of the solitary tract (Wolak et al., 2003). In contrast, Y2R is mainly located presynaptically and can be activated by C-terminal fragments of NPY (Widdowson, 1993; Gerald et al., 1995; Gehlert and Gackenheimer, 1997). Presynaptically, Y2R acts as an autoreceptor on NPY terminals and inhibits the release of endogenous NPY when activated (Chen et al., 1997; King et al., 1999), thus inhibition of Y2R would result in similar behavioral effects as Y1R-stimulation. Y2R also regulates the release of other neurotransmitters like GABA and glutamate (Greber et al., 1994; Sun et al., 2001). Y2R is widely expressed in multiple brain regions such as the amygdala, hippocampus, cortex, thalamus, lateral septum, olfactory nuclei and striatum (Caberlotto et al., 1998; Stanic et al., 2006). Y5R is found in areas such as the cerebral cortex, striatum, hypothalamus, thalamus and hippocampus (Wolak et al., 2003). Both Y5R and Y1R have been implicated in the orexigenic effects of NPY (Gerald et al., 1996; Nguyen et al., 2012). Y1R, Y2R, and Y5R have been implicated in the anxiolytic action of NPY (Heilig et al., 1993; Tschenett et al., 2003; Sorensen et al., 2004). Y4R is predominantly expressed in the peripheral tissues (Parker and Herzog, 1999). Y6R is a non-functional receptor in most mammals (Matsumoto et al., 1996). Research on Y4R and Y6R is much less extensive and will not be discussed in this review.

### NPY and corticostriatal-limbic circuitry

Most research on the mechanisms behind the behavioral effects of NPY have been focused on the actions of NPY in the amygdala since NPY is anxiolytic. The extended amygdala consists of the CeA, the bed nucleus of the stria terminalis, and NAc shell. NPY administration *i.c.v* or into the CeA is anxiolytic in rodents (Heilig et al., 1993). The anxiolytic effect in the amygdala may be mediated by Y1R or Y2R since microinjection of an Y1R agonist and deletion of Y2R in the CeA is anxiolytic (Heilig et al., 1993; Tasan et al., 2010). Thus, Y1R and Y2R appear to have opposite effects on anxiety which can be explained by their receptor localization. Y2R is primarily found presynaptically where it acts as an autoreceptor to inhibit the release of NPY, so antagonism of the receptor would theoretically promote postsynaptic NPY signaling (Chen et al., 1997; King et al., 1999). NPY colocalizes with GABAergic fibers in the CeA and the output neurons of the CeA are mainly GABAergic MSNs (McDonald and Pearson, 1989). Increased activation of the CeA output neurons seems to be associated with increased fear-related behavior (Mohler, 2012). Y1R and Y2R are both coupled to inhibitory G-proteins (Michel, 1991). Thus, activation of Y1R on postsynaptic CeA MSNs and inhibition of presynaptic Y2R on GABAergic terminals could theoretically inhibit the output of the CeA and reduce anxiety (Gilpin et al., 2011). The effects of NPY on CeA activity may also affect behaviors related to AUD since lesioning the CeA reduces alcohol consumption (Moller et al., 1997). It is theorized that acute alcohol increases GABA transmission in the CeA which reduces anxiety, but chronic alcohol may have the opposite effect (Kang-Park et al., 2013). Thus, an Y1R agonist and an Y2R antagonist would be expected to have similar anxiolytic effects as acute alcohol in the CeA (Figure 6). The extended amygdala also plays an important role in alcohol reinforcement and reinstatement, so NPY may affect other alcohol-related behaviors through its actions in the amygdala. Here we will discuss how NPY, especially in the CeA, may reduce alcohol consumption.

### NPY and alcohol in humans

A number of human genetic studies have implicated NPY-related genes in AUD. One of the largest studies identified associations between SNPs in the *NPY2* and *NPY5* receptor genes with alcohol withdrawal symptoms (Wetherill et al., 2008). Other studies have identified SNPs in *NPY* to be associated with alcohol dependence or higher levels of alcohol consumption (Karvonen et al., 1998; Lappalainen et al., 2002; Bhaskar et al., 2013). In humans, a microarray study using postmortem tissue showed that *NPY* expression was reduced in the frontal and motor cortices of chronic alcohol drinkers relative to controls (Mayfield et al., 2002). These studies suggest that a disruption in NPY signaling may increase the risk for developing AUD in humans.

### NPY and alcohol in animal models

Research with alcohol-preferring rat strains suggests that NPY signaling may play an essential role in their alcohol-related behaviors. In two different alcohol preferring rat strains, P rats and HAD rats, baseline NPY levels were lower in the amygdala compared to control rats, suggesting that reduced NPY in the amygdala may promote increased alcohol intake (Ehlers et al., 1998; Hwang et al., 1999). Consistently, genetic linkage studies of P and NP rats identified a QTL on chromosome 4 that was linked to differences alcohol consumption levels and included the NPY gene (Carr et al., 1998). Later, congenic strains created from inbreeding P and NP lines of rats confirmed that the identified QTL from P rats partially explained their increased alcohol consumption and reduced NPY levels (Carr et al., 2006; Liang et al., 2010). These studies suggest that NPY signaling regulates alcohol-related behaviors, especially in the amygdala and cortex.

Conversely, alcohol can affect brain NPY levels. Repeated alcohol consumption or exposure has been shown to decrease NPY in the cortex, amygdala or hypothalamus (Roy and Pandey, 2002; Walker et al., 2010; Sparrow et al., 2012). Another study demonstrated that acute alcohol administration (1 g/kg) or 9 days of voluntary alcohol consumption increased NPY levels in the central and medial amygdala in P rats, but not NP rats (Pandey et al., 2005). In the same study, cAMP response element-binding protein (CREB) activity was quantified since NPY is a CREB-regulated gene. Both alcohol treatments increased CREB activity in P rats, which suggests that acute alcohol-induced increase in NPY may be due to alcohol-mediated activation of CREB (Pandey et al., 2005). One hypothesis that could be formed from these studies is that low doses of alcohol may be consumed to increase NPY to baseline levels. Decreased NPY in the amygdala may result from genetic differences or alcohol withdrawal. Reduced amygdala NPY has been linked to increased anxiety (Pandey et al., 2005) and since low doses of alcohol may increase NPY, alcohol-induced NPY expression may be a possible mechanism for the anxiolytic effect of alcohol.

Animal studies indicate that reduced NPY signaling enhances alcohol consumption. For example, NPY KO mice consumed more alcohol and NPY overexpression lowered alcohol consumption relative to wildtype mice in a two-bottle choice drinking paradigm (Table 1; Thiele et al., 1998). Also, NPY KO mice were less sensitive and NPY overexpressing mice were more sensitive to the sedative effects of alcohol, suggesting that decreased NPY signaling increases tolerance to alcohol intoxication, which could increase drinking (Thiele et al., 1998). Moreover, Y1R KO mice consumed more alcohol, whereas Y2R KO mice drank less alcohol than wildtype controls in a two-bottle choice paradigm (Table 1; Thiele et al., 2002, 2004). The opposing effects of these receptors probably reflect the function of Y2R as a presynaptic autoreceptor. Y5R KO mice appear to drink similar amounts of alcohol as wildtype mice, suggesting that Y5R does not may a major role in AUD (Table 1; Thiele et al., 2000). Pharmacological studies have shown that NPY reduces alcohol consumption, but NPY appears to be effective only in alcohol-preferring rodents or alcohol-exposed animals. For example, *i.c.v.* administration of NPY reduced alcohol intake in a 2 h limited access paradigm in P and HAD rats, but not in NP, LAD or Wistar rats (Badia-Elder et al., 2001, 2003). Central administration of NPY also reduced alcohol drinking-in-the-dark by alcohol-preferring C57BL/6J mice (Sparrow et al., 2012). Other studies showed that *i.c.v.* administration of NPY reduced alcohol consumption (in a limited access and sipper tube operant administration paradigm) in Wistar rats that were previously exposed to alcohol vapor (Thorsell et al., 2005a,b). Another study demonstrated that *i.c.v.* administered NPY more effectively reduced alcohol consumption in P rats that have undergone a period of withdrawal (Gilpin et al., 2003). These latter studies suggest that NPY may reduce drinking by suppressing negative reinforcement for alcohol. Similar results have been obtained with drugs targeting NPY receptors. The Y2R antagonist BIIE0246 suppressed alcohol self-administration in Wistar rats that had been previously exposed to ethanol vapor (Rimondini et al., 2005). In another study, an Y1R agonist and Y2R antagonist reduced alcohol drinking-in-the-dark in C57BL/6J mice (Sparrow et al., 2012). Interestingly, systemic administration of an Y2R antagonist was shown to reverse increases in anxiety-like behavior after alcohol withdrawal, again implying that NPY signaling may regulate negative reinforcement (Cippitelli et al., 2011). Y5R antagonists have been shown to decrease or to have no effect on alcohol self-administration which supports the previous finding that genetic deletion of Y5R does not alter alcohol drinking in mice (Thiele et al., 2000; Schroeder et al., 2003, 2005).

Pharmacological and genetic studies suggest that NPY signaling in the amygdala may be a key mechanism behind the anti-drinking effects of NPY. NPY infusions into the CeA reduced alcohol self-administration in Wistar rats that had been on an alcohol-containing diet, but had no effect in rats that were on an isocaloric diet (non-dependent rats; Gilpin et al., 2008). CeA NPY also reduced two-bottle choice alcohol consumption in P rats that had been previously consuming alcohol (Pandey et al., 2005). Furthermore, viral-mediated overexpression of NPY in the CeA decreased alcohol drinking specifically in anxious Long Evans rats (Primeaux et al., 2006). These studies imply that chronic alcohol exposure or heightened anxiety may increase sensitivity to the anti-drinking effects of NPY in the CeA. The effect of NPY in the CeA on alcohol drinking is likely mediated by its actions on Y2R or Y1R. There is some evidence that NPY acts on presynaptic Y2R in the CeA and inhibits alcohol mediated increases in GABAergic transmission, however this mechanism is controversial since systemic administration of Y2R antagonists reduce alcohol consumption in rodents (Roberto et al., 2003; Gilpin et al., 2011). Thus, further work is necessary to elucidate the actions of NPY and alcohol in the amygdala.

### Summary

Preclinical studies indicate that reduced NPY signaling, especially in the CeA, may increase susceptibility for developing AUD. Therefore, Y1R agonists and Y2R antagonists (since Y2R acts as an autoreceptor) seem promising for the treatment of heavy drinking that is driven by anxiety or negative reinforcement. However, very little is known about the mechanism behind the anti-drinking effects of Y1R agonists and Y2R antagonists. Microinjection of Y1R or Y2R-selective pharmacological agents into the amygdala has yielded conflicting results in regard to alcohol consumption. Considering that Y1R and Y2R are expressed in other brain regions within corticostriatal-limbic circuitry, more work is needed to determine the mechanism behind the therapeutic effect of these drugs.

## Discussion and future direction

Accumulating preclinical and early clinical work suggests that drugs targeting gut-brain peptide signaling are promising therapeutics for the treatment of AUD. Before moving drugs into clinical trials, however, it is essential to validate the therapeutic potential in humans through in-depth behavioral tests and translational research, which includes pharmacodynamic and pharmacokinetic studies in larger animal models. The gut-brain peptides highlighted in this review so far appear to effectively reduce particular addictive behaviors. AUD is a complex heterogeneous disorder. There are numerous reasons why people begin drinking and why they cannot control their use. Comorbid psychiatric and neurological disorders, such as anxiety or chronic pain, may increase propensity to drink and promote relapse. Here, we discussed evidence for why drugs targeting SP and NPY may be useful for the treatment of alcohol dependence with comorbid anxiety. NTS2 or NTS1 agonists may be useful for treating alcohol dependence with comorbid pain-related problems or psychosis. GHS-R1a antagonists have been implicated in alcohol craving, while GLP-1R agonists may be particularly effective in heavy drinkers. For future studies, it will be important to identify subgroups of individuals with AUD that would receive the most benefit and least side effects from potential peptide therapeutics for AUD. Although a daunting task, individualized treatment for AUD seems to be the next frontier for the field. The identification of biomarkers and longitudinal studies will help to demonstrate whether these peptides play a major role in AUD.

## Author contributions

Chelsea A. Vadnie and David J. Hinton constructed the figures. Chelsea A. Vadnie, Jun Hyun Park, Noha Abdel Gawad, Ada Man Choi Ho, David J. Hinton, and Doo-Sup Choi wrote the manuscript.

### Conflict of interest statement

The authors declare that the research was conducted in the absence of any commercial or financial relationships that could be construed as a potential conflict of interest.

## Abbreviations

a.a.: amino-acid
AA: alcohol-accepting
ANA: alcohol-non-accepting
AUD: alcohol use disorder
BBB: blood-brain barrier
CeA: central nucleus of the amygdala
CNS: central nervous system
CPP: conditioned place preference
CREB: cAMP response element-binding protein
D2R: dopamine D2 receptor
DLS: dorsolateral
DMS: dorsomedial
DPP-4: dipeptidyl peptidase-4
Ex4: exendin-4
fMRI: functional magnetic resonance imaging
GHS-R1a: growth hormone secretagogue receptor 1a
GI: gastrointestinal
GLP-1: glucagon-like peptide 1
GLP-1R: glucagon-like peptide 1 receptor
GOAT: ghrelin O-acyltransferase
HAD: high-alcohol-preferring
HPA: hypothalamic-adrenal-pituitary
LAD: low-alcohol-preferring
LDTg: laterodorsal tegmental area
LS: long-sleep
MSNs: medium spiny neurons
NAc: nucleus accumbens
NK1R: neurokinin 1 receptor
NK2R: neurokinin 2 receptor
NK3R: neurokinin 3 receptor
NP: alcohol-non-preferring
NPY: neuropeptide Y
NT: neurotensin
NTS1: neurotensin receptor type 1
NTS2: neurotensin receptor type 2
OCDS: Obsessive Compulsive Drinking Scale
P: alcohol-preferring
PACS: Pennsylvania Alcohol Craving Scores
PFC: prefrontal cortex
QTL: quantitative trait loci
REM: rapid eye movement sleep
SN: substantia nigra
SNPs: single-nucleotide polymorphisms
SP: substance P
SS: short-sleep
WHP: Warsaw high-preferring
WLP: Warsaw low-preferring
VTA: ventral tegmental area.

## References

[B1] Abizaid A. Liu Z.-W. Andrews Z. B. Shanabrough M. Borok E. Elsworth J. D. (2006). Ghrelin modulates the activity and synaptic input organization of midbrain dopamine neurons while promoting appetite. J. Clin. Invest. 116, 3229–3239 10.1172/JCI29867 17060947 PMC1618869

[B2] Addolorato G. Capristo E. Leggio L. Ferrulli A. Abenavoli L. Malandrino N. (2006). Relationship between ghrelin levels, alcohol craving, and nutritional status in current alcoholic patients. Alcohol. Clin. Exp. Res. 30, 1933–1937 10.1111/j.1530-0277.2006.00238.x 17067359

[B3] Agrawal A. Lynskey M. T. Todorov A. A. Schrage A. J. Littlefield A. K. Grant J. D. (2011). A candidate gene association study of alcohol consumption in young women. Alcohol. Clin. Exp. Res. 35, 550–558 10.1111/j.1530-0277.2010.01372.x 21143251 PMC3239405

[B4] Alexander M. J. Leeman S. E. (1998). Widespread expression in adult rat forebrain of mRNA encoding high-affinity neurotensin receptor. J. Comp. Neurol. 402, 475–500 10.1002/(SICI)1096-9861(19981228)402:4<475::AID-CNE4>3.0.CO;2-9 9862322

[B5] Alhadeff A. L. Rupprecht L. E. Hayes M. R. (2012). GLP-1 neurons in the nucleus of the solitary tract project directly to the ventral tegmental area and nucleus accumbens to control for food intake. Endocrinology 153, 647–658 10.1210/en.2011-1443 22128031 PMC3275387

[B6] Alvarez-Crespo M. Skibicka K. P. Farkas I. Molnar C. S. Egecioglu E. Hrabovszky E. (2012). The amygdala as a neurobiological target for ghrelin in rats: neuroanatomical, electrophysiological and behavioral evidence. PLoS ONE 7:e46321 10.1371/journal.pone.0046321 23071554 PMC3468604

[B7] Anderberg R. H. Anefors C. Bergquist F. Nissbrandt H. Skibicka K. P. (2014). Dopamine signaling in the amygdala, increased by food ingestion and GLP-1, regulates feeding behavior. Physiol. Behav. [Epub ahead of print]. 10.1016/j.physbeh.2014.02.026 24560840

[B8] Arnold M. Mura A. Langhans W. Geary N. (2006). Gut vagal afferents are not necessary for the eating-stimulatory effect of intraperitoneally injected ghrelin in the rat. J. Neurosci. 26, 11052–11060 10.1523/JNEUROSCI.2606-06.2006 17065447 PMC6674670

[B9] Badaoui A. De Saeger C. Duchemin J. Gihousse D. de Timary P. Stärkel P. (2008). Alcohol dependence is associated with reduced plasma and fundic ghrelin levels. Eur. J. Clin. Invest. 38, 397–403 10.1111/j.1365-2362.2008.01947.x 18422979

[B10] Badia-Elder N. E. Gilpin N. W. Stewart R. B. (2007). Neuropeptide Y modulation of ethanol intake: effects of ethanol drinking history and genetic background. Peptides 28, 339–344 10.1016/j.peptides.2006.07.028 17187900

[B11] Badia-Elder N. E. Stewart R. B. Powrozek T. A. Murphy J. M. Li T. K. (2003). Effects of neuropeptide Y on sucrose and ethanol intake and on anxiety-like behavior in high alcohol drinking (HAD) and low alcohol drinking (LAD) rats. Alcohol. Clin. Exp. Res. 27, 894–899 10.1111/j.1530-0277.2003.tb04413.x 12824809

[B12] Badia-Elder N. E. Stewart R. B. Powrozek T. A. Roy K. F. Murphy J. M. Li T. K. (2001). Effect of neuropeptide Y (NPY) on oral ethanol intake in Wistar, alcohol-preferring (P), and -nonpreferring (NP) rats. Alcohol. Clin. Exp. Res. 25, 386–390 10.1111/j.1530-0277.2001.tb02225.x 11290849

[B13] Bahi A. Tolle V. Fehrentz J.-A. Brunel L. Martinez J. Tomasetto C.-L. (2013). Ghrelin knockout mice show decreased voluntary alcohol consumption and reduced ethanol-induced conditioned place preference. Peptides 43, 48–55 10.1016/j.peptides.2013.02.008 23428971

[B14] Banks W. A. Tschop M. Robinson S. M. Heiman M. L. (2002). Extent and direction of ghrelin transport across the blood-brain barrier is determined by its unique primary structure. J. Pharmacol. Exp. Ther. 302, 822–827 10.1124/jpet.102.034827 12130749

[B15] Barnes J. M. Barnes N. M. Costall B. Cox A. J. Domeney A. M. Kelly M. E. (1990). Neurochemical consequences following injection of the substance P analogue, DiMe-C7, into the rat ventral tegmental area. Pharmacol. Biochem. Behav. 37, 839–841 10.1016/0091-3057(90)90572-Y 1709506

[B16] Bauer J. Pedersen A. Scherbaum N. Bening J. Patschke J. Kugel H. (2013). Craving in alcohol-dependent patients after detoxification is related to glutamatergic dysfunction in the nucleus accumbens and the anterior cingulate cortex. Neuropsychopharmacology 38, 1401–1408 10.1038/npp.2013.45 23403696 PMC3682141

[B17] Belknap J. K. Crabbe J. C. Young E. R. (1993). Voluntary consumption of ethanol in 15 inbred mouse strains. Psychopharmacology 112, 503–510 10.1007/BF02244901 7871064

[B18] Bell R. L. Rodd Z. A. Lumeng L. Murphy J. M. McBride W. J. (2006). The alcohol-preferring P rat and animal models of excessive alcohol drinking. Addict. Biol. 11, 270–288 10.1111/j.1369-1600.2005.00029.x 16961759

[B19] Bennett B. Carosone-Link P. Zahniser N. R. Johnson T. E. (2006). Confirmation and fine mapping of ethanol sensitivity quantitative trait loci, and candidate gene testing in the LXS recombinant inbred mice. J. Pharm. Exp. Ther. 319, 299–307 10.1124/jpet.106.103572 16803863

[B20] Bertilsson G. Patrone C. Zachrisson O. Andersson A. Dannaeus K. Heidrich J. (2008). Peptide hormone exendin−4 stimulates subventricular zone neurogenesis in the adult rodent brain and induces recovery in an animal model of Parkinson's disease. J. Neurosci. Res. 86, 326–338 10.1002/jnr.21483 17803225

[B21] Bhaskar L. V. Thangaraj K. Kumar K. P. Pardhasaradhi G. Singh L. Rao V. R. (2013). Association between neuropeptide Y gene polymorphisms and alcohol dependence: a case-control study in two independent populations. Eur. Addict. Res. 19, 307–313 10.1159/000346679 23652361

[B22] Binder E. B. Kinkead B. Owens M. J. Nemeroff C. B. (2001a). Neurotensin and dopamine interactions. Pharmacol. Rev. 53, 453–486 11734615

[B23] Binder E. B. Kinkead B. Owens M. J. Nemeroff C. B. (2001b). The role of neurotensin in the pathophysiology of schizophrenia and the mechanism of action of antipsychotic drugs. Biol. Psychiatry 50, 856–872 10.1016/S0006-3223(01)01211-2 11743941

[B24] Bissette G. Nemeroff C. B. Loosen P. T. Prange A. J. Jr. Lipton M. A. (1976). Hypothermia and intolerance to cold induced by intracisternal administration of the hypothalamic peptide neurotensin. Nature 262, 607–609 10.1038/262607a0 8728

[B25] Blaine S. Claus E. Harlaar N. Hutchison K. (2013). TACR1 genotypes predict fMRI response to alcohol cues and level of alcohol dependence. Alcohol. Clin. Exp. Res. 37(Suppl. 1), E125–E130 10.1111/j.1530-0277.2012.01923.x 23078527 PMC5996989

[B26] Blomqvist A. G. Herzog H. (1997). Y-receptor subtypes–how many more? Trends Neurosci. 20, 294–298 10.1016/S0166-2236(96)01057-0 9223221

[B27] Boix F. Mattioli R. Adams F. Huston J. P. Schwarting R. K. (1992). Effects of substance P on extracellular dopamine in neostriatum and nucleus accumbens. Eur. J. Pharmacol. 216, 103–107 10.1016/0014-2999(92)90215-P 1382019

[B28] Borroto-Escuela D. O. Ravani A. Tarakanov A. O. Brito I. Narvaez M. Romero-Fernandez W. (2013). Dopamine D2 receptor signaling dynamics of dopamine D2-neurotensin 1 receptor heteromers. Biochem. Biophys. Res. Commun. 435, 140–146 10.1016/j.bbrc.2013.04.058 23624386

[B29] Boudin H. Pelaprat D. Rostene W. Beaudet A. (1996). Cellular distribution of neurotensin receptors in rat brain: immunohistochemical study using an antipeptide antibody against the cloned high affinity receptor. J. Comp. Neurol. 373, 76–89 10.1002/(SICI)1096-9861(19960909)373:1<76::AID-CNE7>3.0.CO;2-A 8876464

[B30] Boules M. Fredrickson P. Richelson E. (2006). Bioactive analogs of neurotensin: focus on CNS effects. Peptides 27, 2523–2533 10.1016/j.peptides.2005.12.018 16882457

[B31] Boules M. Li Z. Smith K. Fredrickson P. Richelson E. (2013). Diverse roles of neurotensin agonists in the central nervous system. Front. Endocrinol (Lausanne) 4:36 10.3389/fendo.2013.00036 23526754 PMC3605594

[B32] Boules M. Liang Y. Briody S. Miura T. Fauq I. Oliveros A. (2010). NT79: a novel neurotensin analog with selective behavioral effects. Brain Res. 1308, 35–46 10.1016/j.brainres.2009.10.050 19874804 PMC2792000

[B33] Bulik C. M. Klump K. L. Thornton L. Kaplan A. S. Devlin B. Fichter M. M. (2004). Alcohol use disorder comorbidity in eating disorders: a multicenter study. J. Clin. Psychiatry 65, 1000–1006 10.4088/JCP.v65n0718 15291691

[B34] Burgevin M. C. Laduron P. M. Quarteronnet D. Chevet T. Castel M. N. (1992). Striatal injection of neurotensin increases tyrosine hydroxylase mRNA in substantia nigra. Ann. N.Y. Acad. Sci. 668, 311–313 10.1111/j.1749-6632.1992.tb27360.x 1361119

[B35] Caberlotto L. Fuxe K. Rimland J. M. Sedvall G. Hurd Y. L. (1998). Regional distribution of neuropeptide Y Y2 receptor messenger RNA in the human post mortem brain. Neuroscience 86, 167–178 10.1016/S0306-4522(98)00039-6 9692752

[B36] Calissendorff J. Danielsson O. Brismar K. Röjdmark S. (2005). Inhibitory effect of alcohol on ghrelin secretion in normal man. Eur. J. Endocrinol. 152, 743–747 10.1530/eje.1.01905 15879360

[B37] Calissendorff J. Danielsson O. Brismar K. Röjdmark S. (2006). Alcohol ingestion does not affect serum levels of peptide YY but decreases both total and octanoylated ghrelin levels in healthy subjects. Metabolism 55, 1625–1629 10.1016/j.metabol.2006.08.003 17142135

[B38] Campbell A. D. Gene Erwin V. (1993). Chronic ethanol administration downregulates neurotensin receptors in long-and short-sleep mice. Pharmacol. Biochem. Behav. 45, 95–106 10.1016/0091-3057(93)90092-8 8100076

[B39] Carr L. G. Foroud T. Bice P. Gobbett T. Ivashina J. Edenberg H. (1998). A quantitative trait locus for alcohol consumption in selectively bred rat lines. Alcohol. Clin. Exp. Res. 22, 884–887 10.1111/j.1530-0277.1998.tb03883.x 9660316

[B40] Carr L. G. Habegger K. Spence J. P. Liu L. Lumeng L. Foroud T. (2006). Development of congenic rat strains for alcohol consumption derived from the alcohol-preferring and nonpreferring rats. Behav. Genet. 36, 285–290 10.1007/s10519-005-9021-z 16470346

[B41] Carraway R. Leeman S. E. (1973). The isolation of a new hypotensive peptide, neurotensin, from bovine hypothalami. J. Biol. Chem. 248, 6854–6861 4745447

[B42] Chapman M. A. See R. E. Bissette G. (1992). Neurotensin increases extracellular striatal dopamine levels *in vivo*. Neuropeptides 22, 175–183 10.1016/0143-4179(92)90160-X 1436404

[B43] Chen J. Nam H. W. Lee M. R. Hinton D. J. Choi S. Kim T. (2010). Altered glutamatergic neurotransmission in the striatum regulates ethanol sensitivity and intake in mice lacking ENT1. Behav. Brain Res. 208, 636–642 10.1016/j.bbr.2010.01.011 20085785 PMC2831139

[B44] Chen X. Dimaggio D. A. Han S. P. Westfall T. C. (1997). Autoreceptor-induced inhibition of neuropeptide Y release from PC-12 cells is mediated by Y2 receptors. Am. J. Physiol. 273, H1737–H1744 10.1152/ajpheart.00135.2013 9362238

[B45] Cippitelli A. Rezvani A. H. Robinson J. E. Eisenberg L. Levin E. D. Bonaventure P. (2011). The novel, selective, brain-penetrant neuropeptide Y Y2 receptor antagonist, JNJ-31020028, tested in animal models of alcohol consumption, relapse, and anxiety. Alcohol 45, 567–576 10.1016/j.alcohol.2010.09.003 21145691

[B46] Cooke J. H. Patterson M. Patel S. R. Smith K. L. Ghatei M. A. Bloom S. R. (2009). Peripheral and central administration of xenin and neurotensin suppress food intake in rodents. Obesity (Silver Spring) 17, 1135–1143 10.1038/oby.2008.652 19214175

[B47] Crabbe J. C. Gray D. K. Young E. R. Janowsky J. S. Rigter H. (1981). Initial sensitivity and tolerance to ethanol in mice: correlations among open field activity, hypothermia, and loss of righting reflex. Behav. Neural Biol. 33, 188–203 10.1016/S0163-1047(81)91625-3 7305813

[B48] Cummings D. E. Purnell J. Q. Frayo R. S. Schmidova K. Wisse B. E. Weigle D. S. (2001). A preprandial rise in plasma ghrelin levels suggests a role in meal initiation in humans. Diabetes 50, 1714–1719 10.2337/diabetes.50.8.1714 11473029

[B49] Cunningham C. L. (1995). Localization of genes influencing ethanol-induced conditioned place preference and locomotor activity in BXD recombinant inbred mice. Psychopharmacology 120, 28–41 10.1007/BF02246142 7480533

[B50] Cunningham C. L. Gremel C. M. Groblewski P. A. (2006). Drug-induced conditioned place preference and aversion in mice. Nat. Protoc. 1, 1662–1670 10.1038/nprot.2006.279 17487149

[B51] Dansky B. S. Brewerton T. D. Kilpatrick D. G. (2000). Comorbidity of bulimia nervosa and alcohol use disorders: results from the national women's study. Int. J. Eat. Disord. 27, 180–190 10.1002/(SICI)1098-108X(200003)27:2<180::AID-EAT6>3.0.CO;2-Z 10657891

[B52] Dar M. S. (1996). Mouse cerebellar GABAB participation in the expression of acute ethanol-induced ataxia and in its modulation by the cerebellar adenosinergic A1 system. Brain Res. Bull. 41, 53–59 10.1016/0361-9230(96)00172-4 8883916

[B53] Date Y. Murakami N. Toshinai K. Matsukura S. Niijima A. Matsuo H. (2002). The role of the gastric afferent vagal nerve in ghrelin-induced feeding and growth hormone secretion in rats. Gastroenterology 123, 1120–1128 10.1053/gast.2002.35954 12360474

[B54] Davis J. F. Schurdak J. D. Magrisso I. J. Mul J. D. Grayson B. E. Pfluger P. T. (2012). Gastric bypass surgery attenuates ethanol consumption in ethanol-preferring rats. Biol. Psychiatry 72, 354–360 10.1016/j.biopsych.2012.01.035 22444202

[B55] de Timary P. Cani P. D. Duchemin J. Neyrinck A. M. Gihousse D. Laterre P. F. (2012). The loss of metabolic control on alcohol drinking in heavy drinking alcohol-dependent subjects. PLoS ONE 7:e38682 10.1371/journal.pone.0038682 22808013 PMC3392266

[B56] Deutch A. Y. Maggio J. E. Bannon M. J. Kalivas P. W. Tam S. Y. Goldstein M. (1985). Substance K and substance P differentially modulate mesolimbic and mesocortical systems. Peptides 6(Suppl. 2), 113–122 10.1016/0196-9781(85)90143-3 2417207

[B57] Dickson S. L. Egecioglu E. Landgren S. Skibicka K. P. Engel J. A. Jerlhag E. (2011). The role of the central ghrelin system in reward from food and chemical drugs. Mol. Cell. Endocrinol. 340, 80–87 10.1016/j.mce.2011.02.017 21354264

[B58] Dickson S. L. Hrabovszky E. Hansson C. Jerlhag E. Alvarez-Crespo M. Skibicka K. P. (2010). Blockade of central nicotine acetylcholine receptor signaling attenuate ghrelin-induced food intake in rodents. Neuroscience 171, 1180–1186 10.1016/j.neuroscience.2010.10.005 20933579

[B59] Dickson S. L. Shirazi R. H. Hansson C. Bergquist F. Nissbrandt H. Skibicka K. P. (2012). The glucagon-like peptide 1 (GLP-1) analogue, exendin-4, decreases the rewarding value of food: a new role for mesolimbic GLP-1 receptors. J. Neurosci. 32, 4812–4820 10.1523/JNEUROSCI.6326-11.2012 22492036 PMC6620919

[B60] Disse E. Bussier A. L. Deblon N. Pfluger P. T. Tschop M. H. Laville M. (2011). Systemic ghrelin and reward: effect of cholinergic blockade. Physiol. Behav. 102, 481–484 10.1016/j.physbeh.2010.12.006 21163280

[B61] Dossat A. M. Lilly N. Kay K. Williams D. L. (2011). Glucagon-like peptide 1 receptors in nucleus accumbens affect food intake. J. Neurosci. 31, 14453–14457 10.1523/JNEUROSCI.3262-11.2011 21994361 PMC3328130

[B62] Dubuc I. Costentin J. Terranova J. P. Barnouin M. C. Soubrie P. Le Fur G. (1994). The nonpeptide neurotensin antagonist, SR 48692, used as a tool to reveal putative neurotensin receptor subtypes. Br. J. Pharmacol. 112, 352–354 10.1111/j.1476-5381.1994.tb13077.x 8075852 PMC1910364

[B63] During M. J. Cao L. Zuzga D. S. Francis J. S. Fitzsimons H. L. Jiao X. (2003). Glucagon-like peptide-1 receptor is involved in learning and neuroprotection. Nat. Med. 9, 1173–1179 10.1038/nm919 12925848

[B64] Ebner K. Rupniak N. M. Saria A. Singewald N. (2004). Substance P in the medial amygdala: emotional stress-sensitive release and modulation of anxiety-related behavior in rats. Proc. Natl. Acad. Sci. U.S.A. 101, 4280–4285 10.1073/pnas.0400794101 15024126 PMC384732

[B65] Egecioglu E. Engel J. A. Jerlhag E. (2013). The glucagon-like peptide 1 analogue, exendin-4, attenuates the rewarding properties of psychostimulant drugs in mice. PLoS ONE 8:e69010 10.1371/journal.pone.0069010 23874851 PMC3712951

[B66] Egecioglu E. Jerlhag E. Salome N. Skibicka K. P. Haage D. Bohlooly Y. M. (2010). Ghrelin increases intake of rewarding food in rodents. Addict. Biol. 15, 304–311 10.1111/j.1369-1600.2010.00216.x 20477752 PMC2901520

[B67] Egecioglu E. Steensland P. Fredriksson I. Feltmann K. Engel J. A. Jerlhag E. (2012). The glucagon-like peptide 1 analogue Exendin-4 attenuates alcohol mediated behaviors in rodents. Psychoneuroendocrinology 38, 1259–1270 10.1016/j.psyneuen.2012.11.009 23219472

[B68] Ehlers C. L. Li T. K. Lumeng L. Hwang B. H. Somes C. Jimenez P. (1998). Neuropeptide Y levels in ethanol-naive alcohol-preferring and nonpreferring rats and in Wistar rats after ethanol exposure. Alcohol. Clin. Exp. Res. 22, 1778–1782 10.1111/j.1530-0277.1998.tb03979.x 9835294

[B69] Ehlers C. L. Somes C. Li T. K. Lumeng L. Kinkead B. Owens M. J. (1999). Neurotensin studies in alcohol naive, preferring and non-preferring rats. Neuroscience 93, 227–236 10.1016/S0306-4522(99)00113-X 10430486

[B70] Elliott P. J. Alpert J. E. Bannon M. J. Iversen S. D. (1986). Selective activation of mesolimbic and mesocortical dopamine metabolism in rat brain by infusion of a stable substance P analogue into the ventral tegmental area. Brain Res. 363, 145–147 10.1016/0006-8993(86)90667-0 2418910

[B71] Elliott P. J. Mason G. S. Stephens-Smith M. Hagan R. M. (1991). Behavioural and biochemical responses following activation of midbrain dopamine pathways by receptor selective neurokinin agonists. Neuropeptides 19, 119–126 10.1016/0143-4179(91)90141-5 1719444

[B72] Erreger K. Davis A. R. Poe A. M. Greig N. H. Stanwood G. D. Galli A. (2012). Exendin-4 decreases amphetamine-induced locomotor activity. Physiol. Behav. 106, 574–578 10.1016/j.physbeh.2012.03.014 22465309 PMC3348974

[B73] Ervin G. N. Birkemo L. S. Nemeroff C. B. Prange A. J. (1981). Neurotensin blocks certain amphetamine-induced behaviours. Nature 291, 73–76 10.1038/291073a0 7231526

[B74] Erwin V. Campbell A. D. Myers R. Womer D. E. (1995). Cross-tolerance between ethanol and neurotensin in mice selectively bred for ethanol sensitivity. Pharmacol. Biochem. Behav. 51, 891–899 10.1016/0091-3057(95)00070-D 7675874

[B75] Erwin V. G. Gehle V. M. Davidson K. Radcliffe R. A. (2001). Confirmation of correlations and common quantitative trait loci between neurotensin receptor density and hypnotic sensitivity to ethanol. Alcohol. Clin. Exp. Res. 25, 1699–1707 10.1111/j.1530-0277.2001.tb02178.x 11781501

[B76] Erwin V. G. Jones B. C. (1989). Comparison of neurotensin levels, receptors and actions in LS/Ibg and SS/Ibg mice. Peptides 10, 435–440 10.1016/0196-9781(89)90055-7 2547208

[B77] Erwin V. G. Jones B. C. (1993). Genetic correlations among ethanol-related behaviors and neurotensin receptors in long sleep (LS) x short sleep (SS) recombinant inbred strains of mice. Behav. Genet. 23, 191–196 10.1007/BF01067424 8390238

[B78] Erwin V. G. Jones B. C. Radcliffe R. (1990). Low doses of ethanol reduce neurotensin levels in discrete brain regions from LS/Ibg and SS/Ibg mice. Alcohol. Clin. Exp. Res. 14, 42–47 10.1111/j.1530-0277.1990.tb00444.x 2178471

[B79] Everitt B. J. Belin D. Economidou D. Pelloux Y. Dalley J. W. Robbins T. W. (2008). Review. Neural mechanisms underlying the vulnerability to develop compulsive drug-seeking habits and addiction. Phil. Trans. R. Soc. B. Biol. Sci. 363, 3125–3135 10.1098/rstb.2008.0089 18640910 PMC2607322

[B80] Fantegrossi W. E. Ko M. C. Woods J. H. Richelson E. (2005). Antinociceptive, hypothermic, hypotensive, and reinforcing effects of a novel neurotensin receptor agonist, NT69L, in rhesus monkeys. Pharmacol. Biochem. Behav. 80, 341–349 10.1016/j.pbb.2004.12.005 15680187

[B81] Farrell M. S. Pei Y. Wan Y. Yadav P. N. Daigle T. L. Urban D. J. (2013). A Galphas DREADD mouse for selective modulation of cAMP production in striatopallidal neurons. Neuropsychopharmacology 38, 854–862 10.1038/npp.2012.251 23303063 PMC3671990

[B82] Fassio A. Evans G. Grisshammer R. Bolam J. P. Mimmack M. Emson P. C. (2000). Distribution of the neurotensin receptor NTS1 in the rat CNS studied using an amino-terminal directed antibody. Neuropharmacology 39, 1430–1442 10.1016/S0028-3908(00)00060-5 10818259

[B83] Ferraro L. Beggiato S. Tomasini M. C. Fuxe K. Tanganelli S. Antonelli T. (2011). Neurotensin regulates cortical glutamate transmission by modulating N-methyl-D-aspartate receptor functional activity: an *in vivo* microdialysis study. J. Neurosci. Res. 89, 1618–1626 10.1002/jnr.22686 21656844

[B84] Ferraro L. O'Connor W. T. Beggiato S. Tomasini M. C. Fuxe K. Tanganelli S. (2012). Striatal NTS1, dopamine D2 and NMDA receptor regulation of pallidal GABA and glutamate release–a dual-probe microdialysis study in the intranigral 6-hydroxydopamine unilaterally lesioned rat. Eur. J. Neurosci. 35, 207–220 10.1111/j.1460-9568.2011.07949.x 22211865

[B85] Ferraro L. Tomasini M. C. Mazza R. Fuxe K. Fournier J. Tanganelli S. (2008). Neurotensin receptors as modulators of glutamatergic transmission. Brain Res. Rev. 58, 365–373 10.1016/j.brainresrev.2007.11.001 18096238

[B86] File S. E. (1997). Anxiolytic action of a neurokinin1 receptor antagonist in the social interaction test. Pharmacol. Biochem. Behav. 58, 747–752 10.1016/S0091-3057(97)90002-2 9329068

[B87] Fitzpatrick K. Winrow C. J. Gotter A. L. Millstein J. Arbuzova J. Brunner J. (2012). Altered sleep and affect in the neurotensin receptor 1 knockout mouse. Sleep 35, 949–956 10.5665/sleep.1958 22754041 PMC3369230

[B88] Frye G. D. Luttinger D. Nemeroff C. B. Vogel R. A. Prange A. J. Jr. Breese G. R. (1981). Modification of the actions of ethanol by centrally active peptides. Peptides 2(Suppl. 1), 99–106 10.1016/0196-9781(81)90063-2 6267562

[B89] Furness J. B. Hunne B. Matsuda N. Yin L. Russo D. Kato I. (2011). Investigation of the presence of ghrelin in the central nervous system of the rat and mouse. Neuroscience 193, 1–9 10.1016/j.neuroscience.2011.07.063 21835225

[B90] Furudono Y. Ando C. Yamamoto C. Kobashi M. Yamamoto T. (2006). Involvement of specific orexigenic neuropeptides in sweetener-induced overconsumption in rats. Behav. Brain Res. 175, 241–248 10.1016/j.bbr.2006.08.031 17010451

[B91] Gadd C. A. Murtra P. De Felipe C. Hunt S. P. (2003). Neurokinin-1 receptor-expressing neurons in the amygdala modulate morphine reward and anxiety behaviors in the mouse. J. Neurosci. 23, 8271–8280 12967989 10.1523/JNEUROSCI.23-23-08271.2003 PMC6740689

[B92] Gehle V. M. Erwin V. (1998). Common quantitative trait loci for alcohol−related behaviors and CNS neurotensin measures: voluntary ethanol consumption. Alcohol. Clin. Exp. Res. 22, 401–408 10.1111/j.1530-0277.1998.tb03666.x 9581646

[B93] Gehlert D. R. Gackenheimer S. L. (1997). Differential distribution of neuropeptide Y Y1 and Y2 receptors in rat and guinea-pig brains. Neuroscience 76, 215–224 10.1016/S0306-4522(96)00340-5 8971773

[B94] George D. T. Gilman J. Hersh J. Thorsell A. Herion D. Geyer C. (2008). Neurokinin 1 receptor antagonism as a possible therapy for alcoholism. Science 319, 1536–1539 10.1126/science.1153813 18276852

[B95] Gerald C. Walker M. W. Criscione L. Gustafson E. L. Batzl-Hartmann C. Smith K. E. (1996). A receptor subtype involved in neuropeptide-Y-induced food intake. Nature 382, 168–171 10.1038/382168a0 8700207

[B96] Gerald C. Walker M. W. Vaysse P. J. He C. Branchek T. A. Weinshank R. L. (1995). Expression cloning and pharmacological characterization of a human hippocampal neuropeptide Y/peptide YY Y2 receptor subtype. J. Biol. Chem. 270, 26758–26761 10.1074/jbc.270.45.26758 7592910

[B97] Gessa G. L. Muntoni F. Collu M. Vargiu L. Mereu G. (1985). Low doses of ethanol activate dopaminergic neurons in the ventral tegmental area. Brain Res. 348, 201–203 10.1016/0006-8993(85)90381-6 2998561

[B98] Gilman J. M. Ramchandani V. A. Davis M. B. Bjork J. M. Hommer D. W. (2008). Why we like to drink: a functional magnetic resonance imaging study of the rewarding and anxiolytic effects of alcohol. J. Neurosci. 28, 4583–4591 10.1523/JNEUROSCI.0086-08.2008 18448634 PMC2730732

[B99] Gilpin N. W. Misra K. Herman M. A. Cruz M. T. Koob G. F. Roberto M. (2011). Neuropeptide Y opposes alcohol effects on gamma-aminobutyric acid release in amygdala and blocks the transition to alcohol dependence. Biol. Psychiatry 69, 1091–1099 10.1016/j.biopsych.2011.02.004 21459365 PMC3090491

[B100] Gilpin N. W. Misra K. Koob G. F. (2008). Neuropeptide Y in the central nucleus of the amygdala suppresses dependence-induced increases in alcohol drinking. Pharmacol. Biochem. Behav. 90, 475–480 10.1016/j.pbb.2008.04.006 18501411 PMC2480495

[B101] Gilpin N. W. Stewart R. B. Murphy J. M. Li T. K. Badia-Elder N. E. (2003). Neuropeptide Y reduces oral ethanol intake in alcohol-preferring (P) rats following a period of imposed ethanol abstinence. Alcohol. Clin. Exp. Res. 27, 787–794 10.1097/01.ALC.0000065723.93234.1D 12766623

[B102] Gnanapavan S. Kola B. Bustin S. A. Morris D. G. McGee P. Fairclough P. (2002). The tissue distribution of the mRNA of ghrelin and subtypes of its receptor, GHS-R, in humans. J. Clin. Endocrinol. Metab. 87, 2988 10.1210/jcem.87.6.8739 12050285

[B103] Goldbloom D. S. Naranjo C. A. Bremner K. E. Hicks L. K. (1992). Eating disorders and alcohol abuse in women. Br. J. Addict. 87, 913–919 10.1111/j.1360-0443.1992.tb01986.x 1525533

[B104] Goldstein D. B. (1972). Relationship of alcohol dose to intensity of withdrawal signs in mice. J. Pharm. Exp. Ther. 180, 203–215 5062297

[B105] Graham D. Erreger K. Galli A. Stanwood G. (2012). GLP-1 analog attenuates cocaine reward. Mol. Psychiatry 18, 961–962 10.1038/mp.2012.141 23089631 PMC3700649

[B106] Gray T. S. Morley J. E. (1986). Neuropeptide Y: anatomical distribution and possible function in mammalian nervous system. Life Sci. 38, 389–401 10.1016/0024-3205(86)90061-5 3003479

[B107] Graybiel A. M. (2000). The basal ganglia. Curr. Biol. 10, R509–R511 10.1016/S0960-9822(00)00593-5 10899013

[B108] Greber S. Schwarzer C. Sperk G. (1994). Neuropeptide Y inhibits potassium-stimulated glutamate release through Y2 receptors in rat hippocampal slices *in vitro*. Br. J. Pharmacol. 113, 737–740 10.1111/j.1476-5381.1994.tb17055.x 7858862 PMC1510458

[B109] Groenewegen H. J. Wright C. I. Beijer A. V. (1996). The nucleus accumbens: gateway for limbic structures to reach the motor system? Prog. Brain Res. 107, 485–511 10.1016/S0079-6123(08)61883-X 8782538

[B110] Guan X. M. Yu H. Palyha O. C. McKee K. K. Feighner S. D. Sirinathsinghji D. J. (1997). Distribution of mRNA encoding the growth hormone secretagogue receptor in brain and peripheral tissues. Brain Res. Mol. Brain Res. 48, 23–29 10.1016/S0169-328X(97)00071-5 9379845

[B111] Gupta S. Warner J. (2008). Alcohol-related dementia: a 21st-century silent epidemic? Br. J Psychiatry 193, 351–353 10.1192/bjp.bp.108.051425 18978310

[B112] Hansen L. Deacon C. F. Orskov C. Holst J. J. (1999). Glucagon-like peptide-1-(7-36)amide is transformed to glucagon-like peptide-1-(9-36)amide by dipeptidyl peptidase IV in the capillaries supplying the L cells of the porcine intestine. Endocrinology 140, 5356–5363 10.1210/endo.140.11.7143 10537167

[B113] Hayes M. R. Bradley L. Grill H. J. (2009). Endogenous hindbrain glucagon-like peptide-1 receptor activation contributes to the control of food intake by mediating gastric satiation signaling. Endocrinology 150, 2654–2659 10.1210/en.2008-1479 19264875 PMC2689794

[B114] Heilig M. McLeod S. Brot M. Heinrichs S. C. Menzaghi F. Koob G. F. (1993). Anxiolytic-like action of neuropeptide Y: mediation by Y1 receptors in amygdala, and dissociation from food intake effects. Neuropsychopharmacology 8, 357–363 10.1038/npp.1993.35 8099792

[B115] Herpfer I. Lieb K. (2005). Substance P receptor antagonists in psychiatry: rationale for development and therapeutic potential. CNS Drugs 19, 275–293 10.2165/00023210-200519040-00001 15813642

[B116] Hill R. (2000). NK1 (substance P) receptor antagonists–why are they not analgesic in humans? Trends Pharmacol. Sci. 21, 244–246 10.1016/S0165-6147(00)01502-9 10871891

[B117] Hinton D. J. Lee M. R. Jacobson T. L. Mishra P. K. Frye M. A. Mrazek D. A. (2012). Ethanol withdrawal-induced brain metabolites and the pharmacological effects of acamprosate in mice lacking ENT1. Neuropharmacology 62, 2480–2488 10.1016/j.neuropharm.2012.02.022 22616110 PMC3531991

[B118] Holst B. Cygankiewicz A. Jensen T. H. Ankersen M. Schwartz T. W. (2003). High constitutive signaling of the ghrelin receptor–identification of a potent inverse agonist. Mol. Endocrinol. 17, 2201–2210 10.1210/me.2003-0069 12907757

[B119] Huston J. P. Hasenohrl R. U. (1995). The role of neuropeptides in learning: focus on the neurokinin substance P. Behav. Brain Res. 66, 117–127 10.1016/0166-4328(94)00132-Y 7538769

[B120] Hwa L. S. Chu A. Levinson S. A. Kayyali T. M. Debold J. F. Miczek K. A. (2011). Persistent escalation of alcohol drinking in C57BL/6J mice with intermittent access to 20% ethanol. Alcohol. Clin. Exp. Res. 35, 1938–1947 10.1111/j.1530-0277.2011.01545.x 21631540 PMC3166538

[B121] Hwang B. H. Zhang J. K. Ehlers C. L. Lumeng L. Li T. K. (1999). Innate differences of neuropeptide Y (NPY) in hypothalamic nuclei and central nucleus of the amygdala between selectively bred rats with high and low alcohol preference. Alcohol. Clin. Exp. Res. 23, 1023–1030 10.1111/j.1530-0277.1999.tb04220.x 10397286

[B122] Isacson R. Nielsen E. Dannaeus K. Bertilsson G. Patrone C. Zachrisson O. (2011). The glucagon-like peptide 1 receptor agonist exendin-4 improves reference memory performance and decreases immobility in the forced swim test. Eur. J. Pharmacol. 650, 249–255 10.1016/j.ejphar.2010.10.008 20951130

[B123] Jerlhag E. (2008). PRECLINICAL STUDY: systemic administration of ghrelin induces conditioned place preference and stimulates accumbal dopamine. Addict. Biol. 13, 358–363 10.1111/j.1369-1600.2008.00125.x 18782383

[B124] Jerlhag E. Egecioglu E. Dickson S. L. Andersson M. Svensson L. Engel J. A. (2006). Ghrelin stimulates locomotor activity and accumbal dopamine-overflow via central cholinergic systems in mice: implications for its involvement in brain reward. Addict. Biol. 11, 45–54 10.1111/j.1369-1600.2006.00002.x 16759336

[B125] Jerlhag E. Egecioglu E. Dickson S. L. Douhan A. Svensson L. Engel J. A. (2007). Ghrelin administration into tegmental areas stimulates locomotor activity and increases extracellular concentration of dopamine in the nucleus accumbens. Addict. Biol. 12, 6–16 10.1111/j.1369-1600.2006.00041.x 17407492

[B126] Jerlhag E. Egecioglu E. Dickson S. L. Engel J. A. (2010). Ghrelin receptor antagonism attenuates cocaine-and amphetamine-induced locomotor stimulation, accumbal dopamine release, and conditioned place preference. Psychopharmacology 211, 415–422 10.1007/s00213-010-1907-7 20559820 PMC2908453

[B127] Jerlhag E. Egecioglu E. Dickson S. L. Engel J. A. (2011a). Glutamatergic regulation of ghrelin-induced activation of the mesolimbic dopamine system. Addict. Biol. 16, 82–91 10.1111/j.1369-1600.2010.00231.x 20579004 PMC3015055

[B128] Jerlhag E. Egecioglu E. Dickson S. L. Svensson L. Engel J. A. (2008). Alpha-conotoxin MII-sensitive nicotinic acetylcholine receptors are involved in mediating the ghrelin-induced locomotor stimulation and dopamine overflow in nucleus accumbens. Eur. Neuropsychopharmacol. 18, 508–518 10.1016/j.euroneuro.2008.02.006 18343642

[B129] Jerlhag E. Egecioglu E. Landgren S. Salomé N. Heilig M. Moechars D. (2009). Requirement of central ghrelin signaling for alcohol reward. Proc. Natl. Acad. Sci.U.S.A. 106, 11318–11323 10.1073/pnas.0812809106 19564604 PMC2703665

[B130] Jerlhag E. Ivanoff L. Vater A. Engel J. A. (2014). Peripherally circulating ghrelin does not mediate alcohol-induced reward and alcohol intake in rodents. Alcohol. Clin. Exp. Res. 38, 959–968 10.1111/acer.12337 24428428 PMC4112802

[B131] Jerlhag E. Landgren S. Egecioglu E. Dickson S. L. Engel J. A. (2011b). The alcohol-induced locomotor stimulation and accumbal dopamine release is suppressed in ghrelin knockout mice. Alcohol 45, 341–347 10.1016/j.alcohol.2010.10.002 21145690

[B132] Jomphe C. Lemelin P. L. Okano H. Kobayashi K. Trudeau L. E. (2006). Bidirectional regulation of dopamine D2 and neurotensin NTS1 receptors in dopamine neurons. Eur. J. Neurosci. 24, 2789–2800 10.1111/j.1460-9568.2006.05151.x 17116165

[B133] Kalivas P. W. Duffy P. (1990). Effect of acute and daily neurotensin and enkephalin treatments on extracellular dopamine in the nucleus accumbens. J. Neurosci. 10, 2940–2949 1697899 10.1523/JNEUROSCI.10-09-02940.1990 PMC6570231

[B134] Kampov-Polevoy A. Tsoi M. Zvartau E. Neznanov N. Khalitov E. (2001). Sweet liking and family history of alcoholism in hospitalized alcoholic and non-alcoholic patients. Alcohol Alcohol. 36, 165–170 10.1093/alcalc/36.2.165 11259214

[B135] Kampov-Polevoy A. B. Garbutt J. C. Khalitov E. (2003). Family history of alcoholism and response to sweets. Alcohol. Clin. Exp. Res. 27, 1743–1749 10.1097/01.ALC.0000093739.05809.DD 14634489

[B136] Kang-Park M. Kieffer B. L. Roberts A. J. Siggins G. R. Moore S. D. (2013). kappa-Opioid receptors in the central amygdala regulate ethanol actions at presynaptic GABAergic sites. J. Pharmacol. Exp. Ther. 346, 130–137 10.1124/jpet.112.202903 23587526 PMC3684840

[B137] Kanoski S. E. Fortin S. M. Ricks K. M. Grill H. J. (2013). Ghrelin signaling in the ventral hippocampus stimulates learned and motivational aspects of feeding via PI3K-Akt signaling. Biol. Psychiatry 73, 915–923 10.1016/j.biopsych.2012.07.002 22884970 PMC3498600

[B138] Karagiannides I. Torres D. Tseng Y. H. Bowe C. Carvalho E. Espinoza D. (2008). Substance P as a novel anti-obesity target. Gastroenterology 134, 747–755 10.1053/j.gastro.2007.12.032 18325388 PMC2359157

[B139] Karvonen M. K. Pesonen U. Koulu M. Niskanen L. Laakso M. Rissanen A. (1998). Association of a leucine(7)-to-proline(7) polymorphism in the signal peptide of neuropeptide Y with high serum cholesterol and LDL cholesterol levels. Nat. Med. 4, 1434–1437 10.1038/4027 9846584

[B140] Kaur S. Ryabinin A. E. (2010). Ghrelin receptor antagonism decreases alcohol consumption and activation of perioculomotor urocortin-containing neurons. Alcohol. Clin. Exp. Res. 34, 1525–1534 10.1111/j.1530-0277.2010.01237.x 20586761 PMC2929279

[B141] Kenny P. J. (2011). Common cellular and molecular mechanisms in obesity and drug addiction. Nat. Rev. Neurosci. 12, 638–651 10.1038/nrn3105 22011680

[B142] Kim D.-J. Yoon S.-J. Choi B. Kim T.-S. Woo Y. S. Kim W. (2005). Increased fasting plasma ghrelin levels during alcohol abstinence. Alcohol Alcohol. 40, 76–79 10.1093/alcalc/agh108 15520048

[B143] King A. C. de Wit H. McNamara P. J. Cao D. (2011). Rewarding, stimulant, and sedative alcohol responses and relationship to future binge drinking. Arch. Gen. Psychiatry 68, 389–399 10.1001/archgenpsychiatry.2011.26 21464363 PMC4633413

[B144] King P. J. Widdowson P. S. Doods H. N. Williams G. (1999). Regulation of neuropeptide Y release by neuropeptide Y receptor ligands and calcium channel antagonists in hypothalamic slices. J. Neurochem. 73, 641–646 10.1046/j.1471-4159.1999.0730641.x 10428060

[B145] Kinkead B. Nemeroff C. (2006). Novel treatments of schizophrenia: targeting the neurotensin system. CNS Neurol. Disord. Drug Targets 5, 205–218 10.2174/187152706776359655 16611093

[B146] Kinkead B. Nemeroff C. B. (2004). Neurotensin, schizophrenia, and antipsychotic drug action. Int. Rev. Neurobiol. 59, 327–349 10.1016/S0074-7742(04)59013-X 15006494

[B147] Kinzig K. P. D'Alessio D. A. Herman J. P. Sakai R. R. Vahl T. P. Figueiredo H. F. (2003). CNS glucagon-like peptide-1 receptors mediate endocrine and anxiety responses to interoceptive and psychogenic stressors. J. Neurosci. 23, 6163–6170 12867498 10.1523/JNEUROSCI.23-15-06163.2003 PMC6740553

[B148] Kleczkowska P. Lipkowski A. W. (2013). Neurotensin and neurotensin receptors: characteristic, structure-activity relationship and pain modulation–a review. Eur. J. Pharmacol. 716, 54–60 10.1016/j.ejphar.2013.03.004 23500196

[B149] Kojima M. Hosoda H. Date Y. Nakazato M. Matsuo H. Kangawa K. (1999). Ghrelin is a growth-hormone-releasing acylated peptide from stomach. Nature 402, 656–660 10.1038/45230 10604470

[B150] Koob G. F. (2013). Theoretical frameworks and mechanistic aspects of alcohol addiction: alcohol addiction as a reward deficit disorder. Curr. Top Behav. Neurosci. 13, 3–30 10.1007/978-3-642-28720-6_129 21744309 PMC3448980

[B151] Koob G. F. Volkow N. D. (2010). Neurocircuitry of addiction. Neuropsychopharmacology 35, 217–238 10.1038/npp.2009.110 19710631 PMC2805560

[B152] Koopmann A. von der Goltz C. Grosshans M. Dinter C. Vitale M. Wiedemann K. (2012). The association of the appetitive peptide acetylated ghrelin with alcohol craving in early abstinent alcohol dependent individuals. Psychoneuroendocrinology 37, 980–986 10.1016/j.psyneuen.2011.11.005 22172639

[B153] Kornetsky C. (1985). Brain-stimulation reward: a model for the neuronal bases for drug-induced euphoria. NIDA Res. Monogr. 62, 30–50 3935931

[B154] Korotkova T. M. Sergeeva O. A. Eriksson K. S. Haas H. L. Brown R. E. (2003). Excitation of ventral tegmental area dopaminergic and nondopaminergic neurons by orexins/hypocretins. J. Neurosci. 23, 7–11 12514194 10.1523/JNEUROSCI.23-01-00007.2003 PMC6742159

[B155] Koschatzky S. Gmeiner P. (2012). Selective agonists for dopamine/neurotensin receptor heterodimers. ChemMedChem 7, 509–514 10.1002/cmdc.201100499 22213714

[B156] Koschatzky S. Tschammer N. Gmeiner P. (2011). Cross-receptor interactions between dopamine D2L and neurotensin NTS1 receptors modulate binding affinities of dopaminergics. ACS Chem. Neurosci.2, 308–316 10.1021/cn200020y 22778874 PMC3369761

[B157] Kraus T. Schanze A. Gröschl M. Bayerlein K. Hillemacher T. Reulbach U. (2005). Ghrelin levels are increased in alcoholism. Alcohol. Clin. Exp. Res. 29, 2154–2157 10.1097/01.alc.0000191753.82554.7e 16385185

[B158] Kufahl P. R. Zavala A. R. Singh A. Thiel K. J. Dickey E. D. Joyce J. N. (2009). c-Fos expression associated with reinstatement of cocaine-seeking behavior by response-contingent conditioned cues. Synapse 63, 823–835 10.1002/syn.20666 19533625 PMC2748778

[B159] Kwako L. E. George D. T. Schwandt M. L. Spagnolo P. A. Momenan R. Hommer D. W. (2014). The neurokinin-1 receptor antagonist aprepitant in co-morbid alcohol dependence and posttraumatic stress disorder: a human experimental study. Psychopharmacology. [Epub ahead of print]. 10.1007/s00213-014-3665-4 25030801 PMC4512162

[B160] Landgren S. Engel J. A. Hyytia P. Zetterberg H. Blennow K. Jerlhag E. (2011). Expression of the gene encoding the ghrelin receptor in rats selected for differential alcohol preference. Behav. Brain Res. 221, 182–188 10.1016/j.bbr.2011.03.003 21392542

[B161] Landgren S. Jerlhag E. Hallman J. Oreland L. Lissner L. Strandhagen E. (2010). Genetic variation of the ghrelin signaling system in females with severe alcohol dependence. Alcohol. Clin. Exp. Res. 34, 1519–1524 10.1111/j.1530-0277.2010.01236.x 20586762

[B162] Landgren S. Jerlhag E. Zetterberg H. Gonzalez-Quintela A. Campos J. Olofsson U. (2008). Association of pro-ghrelin and GHS-R1A gene polymorphisms and haplotypes with heavy alcohol use and body mass. Alcohol. Clin. Exp. Res. 32, 2054–2061 10.1111/j.1530-0277.2008.00793.x 18828808

[B163] Landgren S. Simms J. A. Hyytiä P. Engel J. A. Bartlett S. E. Jerlhag E. (2012). Ghrelin receptor (GHS−R1A) antagonism suppresses both operant alcohol self−administration and high alcohol consumption in rats. Addict. Biol. 17, 86–94 10.1111/j.1369-1600.2010.00280.x 21309944

[B164] Lappalainen J. Kranzler H. R. Malison R. Price L. H. Van Dyck C. Rosenheck R. A. (2002). A functional neuropeptide Y Leu7Pro polymorphism associated with alcohol dependence in a large population sample from the United States. Arch. Gen. Psychiatry 59, 825–831 10.1001/archpsyc.59.9.825 12215082

[B165] Lee M. R. Hinton D. J. Song J. Y. Lee K. W. Choo C. Johng H. (2010). Neurotensin receptor type 1 regulates ethanol intoxication and consumption in mice. Pharmacol. Biochem. Behav. 95, 235–241 10.1016/j.pbb.2010.01.012 20122953 PMC2830308

[B166] Lee M. R. Hinton D. J. Unal S. S. Richelson E. Choi D. S. (2011). Increased ethanol consumption and preference in mice lacking neurotensin receptor type 2. Alcohol. Clin. Exp. Res. 35, 99–107 10.1111/j.1530-0277.2010.01326.x 21039631 PMC3058519

[B167] Leggio L. Addolorato G. Cippitelli A. Jerlhag E. Kampov−Polevoy A. B. Swift R. M. (2011). Role of feeding−related pathways in alcohol dependence: a focus on sweet preference, NPY, and ghrelin. Alcohol. Clin. Exp. Res. 35, 194–202 10.1111/j.1530-0277.2010.01334.x 21058960

[B168] Leggio L. Ferrulli A. Cardone S. Nesci A. Miceli A. Malandrino N. (2012). Ghrelin system in alcohol−dependent subjects: role of plasma ghrelin levels in alcohol drinking and craving. Addict. Biol. 17, 452–464 10.1111/j.1369-1600.2010.00308.x 21392177 PMC4974482

[B169] Leggio L. Schwandt M. L. Oot E. N. Dias A. A. Ramchandani V. A. (2013). Fasting-induced increase in plasma ghrelin is blunted by intravenous alcohol administration: a within-subject placebo-controlled study. Psychoneuroendocrinology 38, 3085–3091 10.1016/j.psyneuen.2013.09.005 24090583 PMC3844072

[B170] Lessard A. Pickel V. M. (2005). Subcellular distribution and plasticity of neurokinin-1 receptors in the rat substantia nigra and ventral tegmental area. Neuroscience 135, 1309–1323 10.1016/j.neuroscience.2005.07.025 16165296

[B171] Li T. K. Lumeng L. Doolittle D. P. (1993). Selective breeding for alcohol preference and associated responses. Behav. Genet. 23, 163–170 10.1007/BF01067421 8099788

[B172] Li T. K. Lumeng L. McBride W. J. Waller M. B. (1979). Progress toward a voluntary oral consumption model of alcoholism. Drug Alcohol Depend. 4, 45–60 10.1016/0376-8716(79)90040-1 41697

[B173] Li X. M. Ferraro L. Tanganelli S. O'Connor W. T. Hasselrot U. Ungerstedt U. (1995). Neurotensin peptides antagonistically regulate postsynaptic dopamine D2 receptors in rat nucleus accumbens: a receptor binding and microdialysis study. J. Neural Transm. Gen. Sect. 102, 125–137 10.1007/BF01276508 8748677

[B174] Li Z. Boules M. Richelson E. (2011). NT69L blocks ethanol-induced increase of dopamine and glutamate levels in striatum of mouse. Neurosci. Lett. 487, 322–324 10.1016/j.neulet.2010.10.048 20974215

[B175] Liang T. Kimpel M. W. McClintick J. N. Skillman A. R. McCall K. Edenberg H. J. (2010). Candidate genes for alcohol preference identified by expression profiling in alcohol-preferring and -nonpreferring reciprocal congenic rats. Genome Biol. 11:R11 10.1186/gb-2010-11-2-r11 20128895 PMC2872871

[B176] Lobo M. K. Nestler E. J. (2011). The striatal balancing act in drug addiction: distinct roles of direct and indirect pathway medium spiny neurons. Front. Neuroanat. 5:41 10.3389/fnana.2011.00041 21811439 PMC3140647

[B177] Lovinger D. M. White G. Weight F. F. (1989). Ethanol inhibits NMDA-activated ion current in hippocampal neurons. Science 243, 1721–1724 10.1126/science.2467382 2467382

[B178] Luttinger D. Frye G. D. Nemeroff C. B. Prange A. J. Jr. (1983). The effects of neurotensin, beta-endorphin, and bombesin on ethanol-induced behaviors in mice. Psychopharmacology (Berl.) 79, 357–363 10.1007/BF00433418 6304802

[B179] Lyons A. M. Lowery E. G. Sparta D. R. Thiele T. E. (2008). Effects of food availability and administration of orexigenic and anorectic agents on elevated ethanol drinking associated with drinking in the dark procedures. Alcohol. Clin. Exp. Res. 32, 1962–1968 10.1111/j.1530-0277.2008.00784.x 18782340 PMC2588472

[B180] Ma H. Huang Y. L. Zhang B. Wang Y. Zhao H. Du H. (2013). Association between neurotensin receptor 1 gene polymorphisms and alcohol dependence in a male Han Chinese population. J. Mol. Neurosci. 51, 408–415 10.1007/s12031-013-0041-5 23743782

[B181] Malik S. McGlone F. Bedrossian D. Dagher A. (2008). Ghrelin modulates brain activity in areas that control appetitive behavior. Cell Metab. 7, 400–409 10.1016/j.cmet.2008.03.007 18460331

[B182] Manberg P. J. Youngblood W. W. Nemeroff C. B. Rossor M. N. Iversen L. L. Prange A. J. Jr. (1982). Regional distribution of neurotensin in human brain. J. Neurochem. 38, 1777–1780 10.1111/j.1471-4159.1982.tb06664.x 7077336

[B183] Mantyh P. W. Hunt S. P. Maggio J. E. (1984). Substance P receptors: localization by light microscopic autoradiography in rat brain using [3H]SP as the radioligand. Brain Res. 307, 147–165 10.1016/0006-8993(84)90470-0 6087984

[B184] Marinelli P. W. Funk D. Juzytsch W. Harding S. Rice K. C. Shaham Y. (2007). The CRF1 receptor antagonist antalarmin attenuates yohimbine-induced increases in operant alcohol self-administration and reinstatement of alcohol seeking in rats. Psychopharmacology 195, 345–355 10.1007/s00213-007-0905-x 17705061

[B185] Matsumoto M. Nomura T. Momose K. Ikeda Y. Kondou Y. Akiho H. (1996). Inactivation of a novel neuropeptide Y/peptide YY receptor gene in primate species. J. Biol. Chem. 271, 27217–27220 10.1074/jbc.271.44.27217 8910290

[B186] Mayfield R. D. Lewohl J. M. Dodd P. R. Herlihy A. Liu J. Harris R. A. (2002). Patterns of gene expression are altered in the frontal and motor cortices of human alcoholics. J. Neurochem. 81, 802–813 10.1046/j.1471-4159.2002.00860.x 12065639

[B187] Mazella J. Botto J.-M. Guillemare E. Coppola T. Sarret P. Vincent J.-P. (1996). Structure, functional expression, and cerebral localization of the levocabastine-sensitive neurotensin/neuromedin N receptor from mouse brain. J. Neurosci. 16, 5613–5620 8795617 10.1523/JNEUROSCI.16-18-05613.1996 PMC6578974

[B188] McDonald A. J. (1998). Cortical pathways to the mammalian amygdala. Prog. Neurobiol. 55, 257–332 10.1016/S0301-0082(98)00003-3 9643556

[B189] McDonald A. J. Pearson J. C. (1989). Coexistence of GABA and peptide immunoreactivity in non-pyramidal neurons of the basolateral amygdala. Neurosci. Lett. 100, 53–58 10.1016/0304-3940(89)90659-9 2569703

[B190] McFarland K. Davidge S. B. Lapish C. C. Kalivas P. W. (2004). Limbic and motor circuitry underlying footshock-induced reinstatement of cocaine-seeking behavior. J. Neurosci. 24, 1551–1560 10.1523/JNEUROSCI.4177-03.2004 14973230 PMC6730472

[B191] McFarland K. Kalivas P. W. (2001). The circuitry mediating cocaine-induced reinstatement of drug-seeking behavior. J. Neurosci. 21, 8655–8663 11606653 10.1523/JNEUROSCI.21-21-08655.2001 PMC6762812

[B192] Merchenthaler I. (1991). Neurons with access to the general circulation in the central nervous system of the rat: a retrograde tracing study with fluoro-gold. Neuroscience 44, 655–662 10.1016/0306-4522(91)90085-3 1721686

[B193] Merchenthaler I. Lane M. Shughrue P. (1999). Distribution of pre−pro−glucagon and glucagon−like peptide−1 receptor messenger RNAs in the rat central nervous system. J. Comp. Neurol. 403, 261–280 10.1002/(SICI)1096-9861(19990111)403:2<261::AID-CNE8>3.0.CO;2-5 9886047

[B194] Metten P. Crabbe J. C. (2005). Alcohol withdrawal severity in inbred mouse (Mus musculus) strains. Behav. Neurosci. 119, 911–925 10.1037/0735-7044.119.4.911 16187819

[B195] Michel M. C. (1991). Receptors for neuropeptide Y: multiple subtypes and multiple second messengers. Trends Pharmacol. Sci. 12, 389–394 10.1016/0165-6147(91)90610-5 1662423

[B196] Mietlicki-Baase E. G. Ortinski P. I. Reiner D. J. Sinon C. G. McCutcheon J. E. Pierce R. C. (2014). Glucagon-like peptide-1 receptor activation in the nucleus accumbens core suppresses feeding by increasing glutamatergic AMPA/kainate signaling. J. Neurosci. 34, 6985–6992 10.1523/JNEUROSCI.0115-14.2014 24828651 PMC4019807

[B197] Mietlicki-Baase E. G. Ortinski P. I. Rupprecht L. E. Olivos D. R. Alhadeff A. L. Pierce R. C. (2013). The food intake-suppressive effects of glucagon-like peptide-1 receptor signaling in the ventral tegmental area are mediated by AMPA/kainate receptors. Am. J. Physiol. Endocrinol. Metab. 305, E1367–E1374 10.1152/ajpendo.00413.2013 24105414 PMC3882373

[B198] Minabe Y. Emori K. Toor A. Stutzmann G. E. Ashby C. R. Jr. (1996). The effect of the acute and chronic administration of CP 96,345, a selective neurokinin1 receptor antagonist, on midbrain dopamine neurons in the rat: a single unit, extracellular recording study. Synapse 22, 35–45 10.1002/(SICI)1098-2396(199601)22:1<35::AID-SYN4>3.0.CO;2-J 8822476

[B199] Mitrovic I. Napier T. C. (1998). Substance P attenuates and DAMGO potentiates amygdala glutamatergic neurotransmission within the ventral pallidum. Brain Res. 792, 193–206 10.1016/S0006-8993(98)00130-9 9593891

[B200] Moghaddam B. Bolinao M. L. (1994). Biphasic effect of ethanol on extracellular accumulation of glutamate in the hippocampus and the nucleus accumbens. Neurosci. Lett. 178, 99–102 10.1016/0304-3940(94)90299-2 7816349

[B201] Mohler H. (2012). The GABA system in anxiety and depression and its therapeutic potential. Neuropharmacology 62, 42–53 10.1016/j.neuropharm.2011.08.040 21889518

[B202] Moller C. Wiklund L. Sommer W. Thorsell A. Heilig M. (1997). Decreased experimental anxiety and voluntary ethanol consumption in rats following central but not basolateral amygdala lesions. Brain Res. 760, 94–101 10.1016/S0006-8993(97)00308-9 9237523

[B203] Moore E. M. Mariani J. N. Linsenbardt D. N. Melon L. C. Boehm S. L. 2nd. (2010). Adolescent C57BL/6J (but not DBA/2J) mice consume greater amounts of limited-access ethanol compared to adults and display continued elevated ethanol intake into adulthood. Alcohol. Clin. Exp. Res. 34, 734–742 10.1111/j.1530-0277.2009.01143.x 20102570 PMC2883811

[B204] Murtra P. Sheasby A. M. Hunt S. P. De Felipe C. (2000). Rewarding effects of opiates are absent in mice lacking the receptor for substance P. Nature 405, 180–183 10.1038/35012069 10821273

[B205] Nakaya Y. Kaneko T. Shigemoto R. Nakanishi S. Mizuno N. (1994). Immunohistochemical localization of substance P receptor in the central nervous system of the adult rat. J. Comp. Neurol. 347, 249–274 10.1002/cne.903470208 7814667

[B206] Naleid A. M. Grace M. K. Cummings D. E. Levine A. S. (2005). Ghrelin induces feeding in the mesolimbic reward pathway between the ventral tegmental area and the nucleus accumbens. Peptides 26, 2274–2279 10.1016/j.peptides.2005.04.025 16137788

[B207] Napier T. C. Mitrovic I. Churchill L. Klitenick M. A. Lu X. Y. Kalivas P. W. (1995). Substance P in the ventral pallidum: projection from the ventral striatum, and electrophysiological and behavioral consequences of pallidal substance P. Neuroscience 69, 59–70 10.1016/0306-4522(95)00218-8 8637633

[B208] Naqvi N. H. Bechara A. (2009). The hidden island of addiction: the insula. Trends Neurosci. 32, 56–67 10.1016/j.tins.2008.09.009 18986715 PMC3698860

[B209] Naslund E. Barkeling B. King N. Gutniak M. Blundell J. E. Holst J. J. (1999). Energy intake and appetite are suppressed by glucagon-like peptide-1 (GLP-1) in obese men. Int. J. Obes. Relat. Metab. Disord. 23, 304–311 10.1038/sj.ijo.0800818 10193877

[B210] Nemeroff C. B. Bissette G. Prange A. J. Jr. Loosen P. T. Barlow T. S. Lipton M. A. (1977). Neurotensin: central nervous system effects of a hypothalamic peptide. Brain Res. 128, 485–496 10.1016/0006-8993(77)90173-1 406965

[B211] Nguyen A. D. Mitchell N. F. Lin S. Macia L. Yulyaningsih E. Baldock P. A. (2012). Y1 and Y5 receptors are both required for the regulation of food intake and energy homeostasis in mice. PLoS ONE 7:e40191 10.1371/journal.pone.0040191 22768253 PMC3387009

[B212] Nikolaus S. Huston J. P. Hasenohrl R. U. (1999). Reinforcing effects of neurokinin substance P in the ventral pallidum: mediation by the tachykinin NK1 receptor. Eur. J. Pharmacol. 370, 93–99 10.1016/S0014-2999(99)00105-3 10323256

[B213] Pandey S. C. Zhang H. Roy A. Xu T. (2005). Deficits in amygdaloid cAMP-responsive element-binding protein signaling play a role in genetic predisposition to anxiety and alcoholism. J. Clin. Invest. 115, 2762–2773 10.1172/JCI24381 16200210 PMC1236671

[B214] Papp M. Vassout A. Gentsch C. (2000). The NK1-receptor antagonist NKP608 has an antidepressant-like effect in the chronic mild stress model of depression in rats. Behav. Brain Res. 115, 19–23 10.1016/S0166-4328(00)00230-8 10996404

[B215] Parker R. M. Herzog H. (1999). Regional distribution of Y-receptor subtype mRNAs in rat brain. Eur. J. Neurosci. 11, 1431–1448 10.1046/j.1460-9568.1999.00553.x 10103138

[B216] Pelaprat D. (2006). Interactions between neurotensin receptors and G proteins. Peptides 27, 2476–2487 10.1016/j.peptides.2006.04.027 16919370

[B217] Peltonen I. Myohanen T. T. Mannisto P. T. (2012). Different interactions of prolyl oligopeptidase and neurotensin in dopaminergic function of the rat nigrostriatal and mesolimbic pathways. Neurochem. Res. 37, 2033–2041 10.1007/s11064-012-0825-y 22729983

[B218] Perello M. Sakata I. Birnbaum S. Chuang J. C. Osborne-Lawrence S. Rovinsky S. A. (2010). Ghrelin increases the rewarding value of high-fat diet in an orexin-dependent manner. Biol. Psychiatry 67, 880–886 10.1016/j.biopsych.2009.10.030 20034618 PMC2854245

[B219] Perfetti R. Merkel P. (2000). Glucagon-like peptide-1: a major regulator of pancreatic beta-cell function. Eur. J. Endocrinol. 143, 717–725 10.1530/eje.0.1430717 11124853

[B220] Perry T. Lahiri D. K. Chen D. Zhou J. Shaw K. T. Egan J. M. (2002). A novel neurotrophic property of glucagon-like peptide 1: a promoter of nerve growth factor-mediated differentiation in PC12 cells. J. Pharmacol. Exp. Ther. 300, 958–966 10.1124/jpet.300.3.958 11861804

[B221] Petkova-Kirova P. Rakovska A. Della Corte L. Zaekova G. Radomirov R. Mayer A. (2008a). Neurotensin modulation of acetylcholine, GABA, and aspartate release from rat prefrontal cortex studied *in vivo* with microdialysis. Brain Res. Bull. 77, 129–135 10.1016/j.brainresbull.2008.04.003 18721670

[B222] Petkova-Kirova P. Rakovska A. Zaekova G. Ballini C. Corte L. D. Radomirov R. (2008b). Stimulation by neurotensin of dopamine and 5-hydroxytryptamine (5-HT) release from rat prefrontal cortex: possible role of NTR1 receptors in neuropsychiatric disorders. Neurochem. Int. 53, 355–361 10.1016/j.neuint.2008.08.010 18835308

[B223] Primeaux S. D. Wilson S. P. Bray G. A. York D. A. Wilson M. A. (2006). Overexpression of neuropeptide Y in the central nucleus of the amygdala decreases ethanol self-administration in “anxious” rats. Alcohol. Clin. Exp. Res. 30, 791–801 10.1111/j.1530-0277.2006.00092.x 16634847

[B224] Prut L. Belzung C. (2003). The open field as a paradigm to measure the effects of drugs on anxiety-like behaviors: a review. Eur. J. Pharmacol. 463, 3–33 10.1016/S0014-2999(03)01272-X 12600700

[B225] Ramalho R. Almeida J. Beltrao M. Pirraco A. Costa R. Sokhatska O. (2013). Substance P antagonist improves both obesity and asthma in a mouse model. Allergy 68, 48–54 10.1111/all.12052 23176443

[B226] Reddy I. A. Stanwood G. D. Galli A. (2013). Moving beyond energy homeostasis: new roles for glucagon-like peptide-1 in food and drug reward. Neurochem. Int. 73, 49–55 10.1016/j.neuint.2013.10.003 24140429 PMC4120871

[B227] Reep R. L. Winans S. S. (1982). Efferent connections of dorsal and ventral agranular insular cortex in the hamster, Mesocricetus auratus. Neuroscience 7, 2609–2635 10.1016/0306-4522(82)90087-2 7155344

[B228] Reyneke L. Russell V. A. Taljaard J. J. (1990). Evidence that the stimulatory effect of neurotensin on dopamine release in rat nucleus accumbens slices is independent of dopamine D2-receptor activation. Brain Res. 534, 188–194 10.1016/0006-8993(90)90128-X 1981481

[B229] Rhodes J. S. Best K. Belknap J. K. Finn D. A. Crabbe J. C. (2005). Evaluation of a simple model of ethanol drinking to intoxication in C57BL/6J mice. Physiol. Behav. 84, 53–63 10.1016/j.physbeh.2004.10.007 15642607

[B230] Rimondini R. Thorsell A. Heilig M. (2005). Suppression of ethanol self-administration by the neuropeptide Y (NPY) Y2 receptor antagonist BIIE0246: evidence for sensitization in rats with a history of dependence. Neurosci. Lett. 375, 129–133 10.1016/j.neulet.2004.10.084 15670655

[B231] Ripley T. L. Gadd C. A. De Felipe C. Hunt S. P. Stephens D. N. (2002). Lack of self-administration and behavioural sensitisation to morphine, but not cocaine, in mice lacking NK1 receptors. Neuropharmacology 43, 1258–1268 10.1016/S0028-3908(02)00295-2 12527475

[B232] Roberto M. Madamba S. G. Moore S. D. Tallent M. K. Siggins G. R. (2003). Ethanol increases GABAergic transmission at both pre-and postsynaptic sites in rat central amygdala neurons. Proc. Natl. Acad. Sci. U.S.A. 100, 2053–2058 10.1073/pnas.0437926100 12566570 PMC149957

[B233] Robledo P. Maldonado R. Koob G. F. (1993). Neurotensin injected into the nucleus accumbens blocks the psychostimulant effects of cocaine but does not attenuate cocaine self-administration in the rat. Brain Res. 622, 105–112 10.1016/0006-8993(93)90808-Z 8242350

[B234] Rogers J. L. See R. E. (2007). Selective inactivation of the ventral hippocampus attenuates cue-induced and cocaine-primed reinstatement of drug-seeking in rats. Neurobiol. Learn. Mem. 87, 688–692 10.1016/j.nlm.2007.01.003 17337218 PMC1896086

[B235] Rostene W. H. Alexander M. J. (1997). Neurotensin and neuroendocrine regulation. Front. Neuroendocrinol. 18, 115–173 10.1006/frne.1996.0146 9101258

[B236] Roy A. Pandey S. C. (2002). The decreased cellular expression of neuropeptide Y protein in rat brain structures during ethanol withdrawal after chronic ethanol exposure. Alcohol. Clin. Exp. Res. 26, 796–803 10.1111/j.1530-0277.2002.tb02607.x 12068247

[B237] Rupniak N. Carlson E. Harrison T. Oates B. Seward E. Owen S. (2000). Pharmacological blockade or genetic deletion of substance P (NK(1)) receptors attenuates neonatal vocalisation in guinea-pigs and mice. Neuropharmacology 39, 1413–1421 10.1016/S0028-3908(00)00052-6 10818257

[B238] Salome N. Haage D. Perrissoud D. Moulin A. Demange L. Egecioglu E. (2009). Anorexigenic and electrophysiological actions of novel ghrelin receptor (GHS-R1A) antagonists in rats. Eur. J. Pharmacol. 612, 167–173 10.1016/j.ejphar.2009.03.066 19356720

[B239] Samson H. H. Pfeffer A. O. Tolliver G. A. (1988). Oral ethanol self-administration in rats: models of alcohol-seeking behavior. Alcohol. Clin. Exp. Res. 12, 591–598 10.1111/j.1530-0277.1988.tb00248.x 3067600

[B240] Sanchis-Segura C. Spanagel R. (2006). REVIEW: behavioural assessment of drug reinforcement and addictive features in rodents: an overview. Addict. Biol. 11, 2–38 10.1111/j.1369-1600.2006.00012.x 16759333

[B241] Santarelli L. Gobbi G. Debs P. C. Sibille E. L. Blier P. Hen R. (2001). Genetic and pharmacological disruption of neurokinin 1 receptor function decreases anxiety-related behaviors and increases serotonergic function. Proc. Natl. Acad. Sci. U.S.A. 98, 1912–1917 10.1073/pnas.98.4.1912 11172050 PMC29356

[B242] Sarret P. Perron A. Stroh T. Beaudet A. (2003). Immunohistochemical distribution of NTS2 neurotensin receptors in the rat central nervous system. J. Comp. Neurol. 461, 520–538 10.1002/cne.10718 12746866

[B243] Saunders B. T. Robinson T. E. (2012). The role of dopamine in the accumbens core in the expression of Pavlovian-conditioned responses. Eur. J. Neurosci. 36, 2521–2532 10.1111/j.1460-9568.2012.08217.x 22780554 PMC3424374

[B244] Schank J. R. (2014). The neurokinin-1 receptor in addictive processes. J. Pharmacol. Exp. Ther. 351, 2–8 10.1124/jpet.113.210799 25038175

[B245] Schank J. R. King C. E. Sun H. Cheng K. Rice K. C. Heilig M. (2014). The role of the neurokinin-1 receptor in stress-induced reinstatement of alcohol and cocaine seeking. Neuropsychopharmacology 39, 1093–1101 10.1038/npp.2013.309 24173499 PMC3957103

[B246] Schank J. R. Pickens C. L. Rowe K. E. Cheng K. Thorsell A. Rice K. C. (2011). Stress-induced reinstatement of alcohol-seeking in rats is selectively suppressed by the neurokinin 1 (NK1) antagonist L822429. Psychopharmacology 218, 111–119 10.1007/s00213-011-2201-z 21340476 PMC3192232

[B247] Schank J. R. Tapocik J. D. Barbier E. Damadzic R. Eskay R. L. Sun H. (2013). Tacr1 gene variation and neurokinin 1 receptor expression is associated with antagonist efficacy in genetically selected alcohol-preferring rats. Biol. Psychiatry 73, 774–781 10.1016/j.biopsych.2012.12.027 23419547 PMC3773538

[B248] Schick R. R. Zimmermann J. P. Vorm Walde T. Schusdziarra V. (2003). Peptides that regulate food intake: glucagon-like peptide 1-(7-36) amide acts at lateral and medial hypothalamic sites to suppress feeding in rats. Am. J. Physiol. Regul. Integr. Comp. Physiol. 284, R1427–R1435 10.1152/ajpregu.00479.2002 12776726

[B249] Schneider E. R. Rada P. Darby R. D. Leibowitz S. F. Hoebel B. G. (2007). Orexigenic peptides and alcohol intake: differential effects of orexin, galanin, and ghrelin. Alcohol. Clin. Exp. Res. 31, 1858–1865 10.1111/j.1530-0277.2007.00510.x 17850217

[B250] Schroeder J. P. Iller K. A. Hodge C. W. (2003). Neuropeptide-Y Y5 receptors modulate the onset and maintenance of operant ethanol self-administration. Alcohol. Clin. Exp. Res. 27, 1912–1920 10.1097/01.ALC.0000098873.80433.BA 14691378

[B251] Schroeder J. P. Overstreet D. H. Hodge C. W. (2005). The neuropeptide-Y Y5 receptor antagonist L-152,804 decreases alcohol self-administration in inbred alcohol-preferring (iP) rats. Alcohol 36, 179–186 10.1016/j.alcohol.2005.10.001 16377459 PMC11606053

[B252] Seneviratne C. Ait-Daoud N. Ma J. Z. Chen G. Johnson B. A. Li M. D. (2009). Susceptibility locus in neurokinin-1 receptor gene associated with alcohol dependence. Neuropsychopharmacology 34, 2442–2449 10.1038/npp.2009.65 19553914 PMC2743763

[B253] Sesack S. R. Grace A. A. (2010). Cortico-Basal Ganglia reward network: microcircuitry. Neuropsychopharmacology 35, 27–47 10.1038/npp.2009.93 19675534 PMC2879005

[B254] Severini C. Improta G. Falconieri-Erspamer G. Salvadori S. Erspamer V. (2002). The tachykinin peptide family. Pharmacol. Rev. 54, 285–322 10.1124/pr.54.2.285 12037144

[B255] Sheikh S. P. Hakanson R. Schwartz T. W. (1989). Y1 and Y2 receptors for neuropeptide Y. FEBS Lett. 245, 209–214 10.1016/0014-5793(89)80223-6 2538360

[B256] Shirazi R. H. Dickson S. L. Skibicka K. P. (2013). Gut peptide GLP-1 and its analogue, Exendin-4, decrease alcohol intake and reward. PLoS ONE 8:e61965 10.1371/journal.pone.0061965 23613987 PMC3628574

[B257] Shults C. W. Quirion R. Chronwall B. Chase T. N. O'Donohue T. L. (1984). A comparison of the anatomical distribution of substance P and substance P receptors in the rat central nervous system. Peptides 5, 1097–1128 10.1016/0196-9781(84)90177-3 6085163

[B258] Skibicka K. P. (2013). The central GLP-1: implications for food and drug reward. Front. Neurosci. 7:181 10.3389/fnins.2013.00181 24133407 PMC3796262

[B259] Skibicka K. P. Dickson S. L. (2011). Ghrelin and food reward: the story of potential underlying substrates. Peptides 32, 2265–2273 10.1016/j.peptides.2011.05.016 21621573

[B260] Skibicka K. P. Hansson C. Alvarez-Crespo M. Friberg P. A. Dickson S. L. (2011). Ghrelin directly targets the ventral tegmental area to increase food motivation. Neuroscience 180, 129–137 10.1016/j.neuroscience.2011.02.016 21335062

[B261] Skibicka K. P. Hansson C. Egecioglu E. Dickson S. L. (2012). Role of ghrelin in food reward: impact of ghrelin on sucrose self-administration and mesolimbic dopamine and acetylcholine receptor gene expression. Addict. Biol. 17, 95–107 10.1111/j.1369-1600.2010.00294.x 21309956 PMC3298643

[B262] Skibicka K. P. Shirazi R. H. Rabasa-Papio C. Alvarez-Crespo M. Neuber C. Vogel H. (2013). Divergent circuitry underlying food reward and intake effects of ghrelin: dopaminergic VTA-accumbens projection mediates ghrelin's effect on food reward but not food intake. Neuropharmacology 73, 274–283 10.1016/j.neuropharm.2013.06.004 23770258

[B263] Slawecki C. J. Jimenéz-Vasquez P. Mathé A. A. Ehlers C. L. (2001). Substance P and neurokinin levels are decreased in the cortex and hypothalamus of alcohol-preferring (P) rats. J. Stud. Alcohol 62, 736 11838908 10.15288/jsa.2001.62.736

[B264] Slawecki C. J. Jiménez-Vasquez P. Mathé A. A. Ehlers C. L. (2005). Effect of ethanol on brain neuropeptides in adolescent and adult rats. J. Stud. Alcohol 66, 46 15830902 10.15288/jsa.2005.66.46

[B265] Sorensen G. Lindberg C. Wortwein G. Bolwig T. G. Woldbye D. P. (2004). Differential roles for neuropeptide Y Y1 and Y5 receptors in anxiety and sedation. J. Neurosci. Res. 77, 723–729 10.1002/jnr.20200 15352219

[B266] Sparrow A. M. Lowery-Gionta E. G. Pleil K. E. Li C. Sprow G. M. Cox B. R. (2012). Central neuropeptide Y modulates binge-like ethanol drinking in C57BL/6J mice via Y1 and Y2 receptors. Neuropsychopharmacology 37, 1409–1421 10.1038/npp.2011.327 22218088 PMC3327846

[B267] Stafford D. Lesage M. G. Glowa J. R. (1998). Progressive-ratio schedules of drug delivery in the analysis of drug self-administration: a review. Psychopharmacology 139, 169–184 10.1007/s002130050702 9784071

[B268] Stanic D. Brumovsky P. Fetissov S. Shuster S. Herzog H. Hokfelt T. (2006). Characterization of neuropeptide Y2 receptor protein expression in the mouse brain. I. Distribution in cell bodies and nerve terminals. J. Comp. Neurol. 499, 357–390 10.1002/cne.21046 16998904

[B269] Steensland P. Simms J. A. Nielsen C. K. Holgate J. Bito-Onon J. J. Bartlett S. E. (2010). The neurokinin 1 receptor antagonist, ezlopitant, reduces appetitive responding for sucrose and ethanol. PLoS ONE 5:e12527 10.1371/journal.pone.0012527 20824145 PMC2931709

[B270] Stewart R. B. Gatto G. J. Lumeng L. Li T. K. Murphy J. M. (1993). Comparison of alcohol-preferring (P) and nonpreferring (NP) rats on tests of anxiety and for the anxiolytic effects of ethanol. Alcohol 10, 1–10 10.1016/0741-8329(93)90046-Q 8095393

[B271] Stolakis V. Kalafatakis K. Botis J. Zarros A. Liapi C. (2010). The regulatory role of neurotensin on the hypothalamic-anterior pituitary axons: emphasis on the control of thyroid-related functions. Neuropeptides 44, 1–7 10.1016/j.npep.2009.09.005 19878995

[B272] Suchankova P. Steensland P. Fredriksson I. Engel J. A. Jerlhag E. (2013). Ghrelin receptor (GHS-R1A) antagonism suppresses both alcohol consumption and the alcohol deprivation effect in rats following long-term voluntary alcohol consumption. PLoS ONE 8:e71284 10.1371/journal.pone.0071284 23977009 PMC3748070

[B273] Sumners C. Phillips M. I. Richards E. M. (1982). Central pressor action of neurotensin in conscious rats. Hypertension 4, 888–893 10.1161/01.HYP.4.6.888 6815078

[B274] Sun Q. Q. Akk G. Huguenard J. R. Prince D. A. (2001). Differential regulation of GABA release and neuronal excitability mediated by neuropeptide Y1 and Y2 receptors in rat thalamic neurons. J. Physiol. 531, 81–94 10.1111/j.1469-7793.2001.0081j.x 11179393 PMC2278458

[B275] Szulc M. Mikolajczak P. L. Geppert B. Wachowiak R. Dyr W. Bobkiewicz-Kozlowska T. (2013). Ethanol affects acylated and total ghrelin levels in peripheral blood of alcohol-dependent rats. Addict. Biol. 18, 689–701 10.1111/adb.12025 23311595

[B276] Szulc P. Zgliczyñski W. Jeske W. Soszyñski P. Rosłonowska E. Nowakowski J. (1991). The effect of oral ethanol ingestion on the diurnal neurotensin secretion in man. Endokrynol. Pol. 43, 264–272 1345566

[B277] Tamiya R. Hanada M. Kawai Y. Inagaki S. Takagi H. (1990). Substance P afferents have synaptic contacts with dopaminergic neurons in the ventral tegmental area of the rat. Neurosci. Lett. 110, 11–15 10.1016/0304-3940(90)90779-9 1691469

[B278] Tasan R. O. Nguyen N. K. Weger S. Sartori S. B. Singewald N. Heilbronn R. (2010). The central and basolateral amygdala are critical sites of neuropeptide Y/Y2 receptor-mediated regulation of anxiety and depression. J. Neurosci. 30, 6282–6290 10.1523/JNEUROSCI.0430-10.2010 20445054 PMC3073168

[B279] Tatemoto K. Carlquist M. Mutt V. (1982). Neuropeptide Y–a novel brain peptide with structural similarities to peptide YY and pancreatic polypeptide. Nature 296, 659–660 10.1038/296659a0 6896083

[B280] Teixeira R. M. Santos A. R. Ribeiro S. J. Calixto J. B. Rae G. A. De Lima T. (1996). Effects of central administration of tachykinin receptor agonists and antagonists on plus-maze behavior in mice. Eur. J. Pharmacol. 311, 7–14 10.1016/0014-2999(96)00390-1 8884230

[B281] Thibault D. Albert P. R. Pineyro G. Trudeau L. E. (2011). Neurotensin triggers dopamine D2 receptor desensitization through a protein kinase C and beta-arrestin1-dependent mechanism. J. Biol. Chem. 286, 9174–9184 10.1074/jbc.M110.166454 21233215 PMC3059057

[B282] Thiele T. E. Koh M. T. Pedrazzini T. (2002). Voluntary alcohol consumption is controlled via the neuropeptide Y Y1 receptor. J. Neurosci. 22, RC208 11826154 10.1523/JNEUROSCI.22-03-j0006.2002 PMC6758511

[B283] Thiele T. E. Marsh D. J. Marie L. S. Bernstein I. L. Palmiter R. D. (1998). Ethanol consumption and resistance are inversely related to neuropeptide Y levels. Nature 396, 366–369 10.1038/24614 9845072

[B284] Thiele T. E. Miura G. I. Marsh D. J. Bernstein I. L. Palmiter R. D. (2000). Neurobiological responses to ethanol in mutant mice lacking neuropeptide Y or the Y5 receptor. Pharmacol. Biochem. Behav. 67, 683–691 10.1016/S0091-3057(00)00413-5 11166058

[B285] Thiele T. E. Naveilhan P. Ernfors P. (2004). Assessment of ethanol consumption and water drinking by NPY Y(2) receptor knockout mice. Peptides 25, 975–983 10.1016/j.peptides.2004.03.009 15203244

[B286] Thorens B. Porret A. Bühler L. Deng S.-P. Morel P. Widmann C. (1993). Cloning and functional expression of the human islet GLP-1 receptor: demonstration that exendin-4 is an agonist and exendin-(9–39) an antagonist of the receptor. Diabetes 42, 1678–1682 10.2337/diab.42.11.1678 8405712

[B287] Thorsell A. Schank J. R. Singley E. Hunt S. P. Heilig M. (2010). Neurokinin-1 receptors (NK1R: s), alcohol consumption, and alcohol reward in mice. Psychopharmacology 209, 103–111 10.1007/s00213-010-1775-1 20112009

[B288] Thorsell A. Slawecki C. J. Ehlers C. L. (2005a). Effects of neuropeptide Y and corticotropin-releasing factor on ethanol intake in Wistar rats: interaction with chronic ethanol exposure. Behav. Brain Res. 161, 133–140 10.1016/j.bbr.2005.01.016 15904720

[B289] Thorsell A. Slawecki C. J. Ehlers C. L. (2005b). Effects of neuropeptide Y on appetitive and consummatory behaviors associated with alcohol drinking in wistar rats with a history of ethanol exposure. Alcohol. Clin. Exp. Res. 29, 584–590 10.1097/01.ALC.0000160084.13148.02 15834223

[B290] Topple A. N. Hunt G. E. McGregor I. S. (1998). Possible neural substrates of beer-craving in rats. Neurosci. Lett. 252, 99–102 10.1016/S0304-3940(98)00574-6 9756331

[B291] Trapp S. Hisadome K. (2011). Glucagon-like peptide 1 and the brain: central actions-central sources? Auton. Neurosci.161, 14–19 10.1016/j.autneu.2010.09.008 20951098

[B292] Tschenett A. Singewald N. Carli M. Balducci C. Salchner P. Vezzani A. (2003). Reduced anxiety and improved stress coping ability in mice lacking NPY-Y2 receptors. Eur. J. Neurosci. 18, 143–148 10.1046/j.1460-9568.2003.02725.x 12859347

[B293] Tschöp M. Smiley D. L. Heiman M. L. (2000). Ghrelin induces adiposity in rodents. Nature 407, 908–913 10.1038/35038090 11057670

[B294] Turton M. D. O'Shea D. Gunn I. Beak S. A. Edwards C. M. Meeran K. (1996). A role for glucagon-like peptide-1 in the central regulation of feeding. Nature 379, 69–72 10.1038/379069a0 8538742

[B295] Us V. E. Gaddum J. H. (1931). An unidentified depressor substance in certain tissue extracts. J. Physiol. 72, 74–87 16994201 10.1113/jphysiol.1931.sp002763 PMC1403098

[B296] Vadnie C. A. Hinton D. J. Choi S. Choi Y. Ruby C. L. Oliveros A. (2014). Activation of neurotensin receptor type 1 attenuates locomotor activity. Neuropharmacology 85, 482–492 10.1016/j.neuropharm.2014.05.046 24929110 PMC4107019

[B297] Valdez G. R. Roberts A. J. Chan K. Davis H. Brennan M. Zorrilla E. P. (2002). Increased ethanol self-administration and anxiety-like behavior during acute ethanol withdrawal and protracted abstinence: regulation by corticotropin-releasing factor. Alcohol. Clin. Exp. Res. 26, 1494–1501 10.1111/j.1530-0277.2002.tb02448.x 12394282

[B298] Vincent J.-P. Mazella J. Kitabgi P. (1999). Neurotensin and neurotensin receptors. Trends Pharmacol. Sci. 20, 302–309 10.1016/S0165-6147(99)01357-7 10390649

[B299] Volkow N. D. Fowler J. S. Wang G. J. Baler R. Telang F. (2009). Imaging dopamine's role in drug abuse and addiction. Neuropharmacology 56(Suppl. 1), 3–8 10.1016/j.neuropharm.2008.05.022 18617195 PMC2696819

[B300] Volkow N. D. Wang G. J. Fowler J. S. Tomasi D. (2012a). Addiction circuitry in the human brain. Annu. Rev. Pharmacol. Toxicol. 52, 321–336 10.1146/annurev-pharmtox-010611-134625 21961707 PMC3477468

[B301] Volkow N. D. Wang G. J. Fowler J. S. Tomasi D. Baler R. (2012b). Food and drug reward: overlapping circuits in human obesity and addiction. Curr. Top. Behav. Neurosci. 11, 1–24 10.1007/7854_2011_169 22016109

[B302] Wachi M. Fujimaki M. Nakamura H. Inazuki G. (1996). Effects of ethanol administration on brain neurotensin−like immunoreactivity in rats. Acta Neurol. Scand. 93, 211–214 10.1111/j.1600-0404.1996.tb00202.x 8741146

[B303] Walker B. M. Drimmer D. A. Walker J. L. Liu T. Mathe A. A. Ehlers C. L. (2010). Effects of prolonged ethanol vapor exposure on forced swim behavior, and neuropeptide Y and corticotropin-releasing factor levels in rat brains. Alcohol 44, 487–493 10.1016/j.alcohol.2010.06.006 20705420 PMC2954060

[B304] Walker N. Lepee-Lorgeoux I. Fournier J. Betancur C. Rostene W. Ferrara P. (1998). Tissue distribution and cellular localization of the levocabastine-sensitive neurotensin receptor mRNA in adult rat brain. Mol. Brain Res. 57, 193–200 10.1016/S0169-328X(98)00074-6 9675417

[B305] Wang R. Boules M. Tiner W. Richelson E. (2004). Effects of repeated injections of the neurotensin analog NT69L on dopamine release and uptake in rat striatum *in vitro*. Brain Res. 1025, 21–28 10.1016/j.brainres.2004.07.069 15464740

[B306] Weiss F. (2005). Neurobiology of craving, conditioned reward and relapse. Curr. Opin. Pharmcol. 5, 9–19 10.1016/j.coph.2004.11.001 15661620

[B307] Wetherill L. Schuckit M. A. Hesselbrock V. Xuei X. Liang T. Dick D. M. (2008). Neuropeptide Y receptor genes are associated with alcohol dependence, alcohol withdrawal phenotypes, and cocaine dependence. Alcohol. Clin. Exp. Res. 32, 2031–2040 10.1111/j.1530-0277.2008.00790.x 18828811 PMC2650441

[B308] Widdowson P. S. (1993). Quantitative receptor autoradiography demonstrates a differential distribution of neuropeptide-Y Y1 and Y2 receptor subtypes in human and rat brain. Brain Res. 631, 27–38 10.1016/0006-8993(93)91182-R 8298993

[B309] Wolak M. L. Dejoseph M. R. Cator A. D. Mokashi A. S. Brownfield M. S. Urban J. H. (2003). Comparative distribution of neuropeptide Y Y1 and Y5 receptors in the rat brain by using immunohistochemistry. J. Comp. Neurol. 464, 285–311 10.1002/cne.10823 12900925

[B310] Woodard G. A. Downey J. Hernandez-Boussard T. Morton J. M. (2011). Impaired alcohol metabolism after gastric bypass surgery: a case-crossover trial. J. Am. Coll. Surg. 212, 209–214 10.1016/j.jamcollsurg.2010.09.020 21183366

[B311] Woolf N. J. (1991). Cholinergic systems in mammalian brain and spinal cord. Prog. Neurobiol. 37, 475–524 10.1016/0301-0082(91)90006-M 1763188

[B312] Wren A. M. Small C. J. Ward H. L. Murphy K. G. Dakin C. L. Taheri S. (2000). The novel hypothalamic peptide ghrelin stimulates food intake and growth hormone secretion. Endocrinology 141, 4325–4328 10.1210/endo.141.11.7873 11089570

[B313] Wurst F. M. Graf I. Ehrenthal H. D. Klein S. Backhaus J. Blank S. (2007). Gender Differences for ghrelin levels in alcohol−dependent patients and differences between alcoholics and healthy controls. Alcohol. Clin. Exp. Res. 31, 2006–2011 10.1111/j.1530-0277.2007.00527.x 17949465

[B314] Yang A. R. Yi H. S. Mamczarz J. June H. L. Jr. Hwang B. H. June H. L. Sr. (2009). Deficits in substance P mRNA levels in the CeA are inversely associated with alcohol-motivated responding. Synapse 63, 972–981 10.1002/syn.20677 19593822 PMC2754587

[B315] Yang J. Brown M. S. Liang G. Grishin N. V. Goldstein J. L. (2008). Identification of the acyltransferase that octanoylates ghrelin, an appetite-stimulating peptide hormone. Cell 132, 387–396 10.1016/j.cell.2008.01.017 18267071

[B316] Yin H. H. Knowlton B. J. (2006). The role of the basal ganglia in habit formation. Nat. Rev. Neurosci. 7, 464–476 10.1038/nrn1919 16715055

[B317] Young A. A. Gedulin B. Bhavsar S. Bodkin N. Jodka C. Hansen B. (1999). Glucose-lowering and insulin-sensitizing actions of exendin-4: studies in obese diabetic (ob/ob, db/db) mice, diabetic fatty Zucker rats, and diabetic rhesus monkeys (*Macaca mulatta*). Diabetes 48, 1026–1034 10.2337/diabetes.48.5.1026 10331407

[B318] Yousseif A. Emmanuel J. Karra E. Millet Q. Elkalaawy M. Jenkinson A. D. (2014). Differential effects of laparoscopic sleeve gastrectomy and laparoscopic gastric bypass on appetite, circulating acyl-ghrelin, peptide YY3-36 and active GLP-1 levels in non-diabetic humans. Obes. Surg. 24, 241–252 10.1007/s11695-013-1066-0 23996294 PMC3890046

[B319] Zhao Z. Yang Y. Walker D. L. Davis M. (2009). Effects of substance P in the amygdala, ventromedial hypothalamus, and periaqueductal gray on fear-potentiated startle. Neuropsychopharmacology 34, 331–340 10.1038/npp.2008.55 18418359 PMC3792658

[B320] Zigman J. M. Jones J. E. Lee C. E. Saper C. B. Elmquist J. K. (2006). Expression of ghrelin receptor mRNA in the rat and the mouse brain. J. Comp. Neurol. 494, 528–548 10.1002/cne.20823 16320257 PMC4524499

[B321] Zimmermann U. S. Buchmann A. Steffin B. Dieterle C. Uhr M. (2007). CLINICAL STUDY: alcohol administration acutely inhibits ghrelin secretion in an experiment involving psychosocial stress. Addict. Biol. 12, 17–21 10.1111/j.1369-1600.2006.00026.x 17407493

